# Exciton Chirality Inversion in Dye Dimers Templated by DNA Holliday Junction

**DOI:** 10.1021/acs.jpclett.2c02721

**Published:** 2022-11-10

**Authors:** Olga A. Mass, Shibani Basu, Lance K. Patten, Ewald A. Terpetschnig, Alexander I. Krivoshey, Anatoliy L. Tatarets, Ryan D. Pensack, Bernard Yurke, William B. Knowlton, Jeunghoon Lee

**Affiliations:** ^†^Micron School of Materials Science & Engineering, ^∥^Department of Electrical & Computer Engineering, and ^⊥^Department of Chemistry and Biochemistry, Boise State University, Boise, Idaho 83725, United States; ‡ SETA BioMedicals, LLC, 2014 Silver Court East, Urbana, Illinois 61801, United States; § SSI “Institute for Single Crystals” of the National Academy of Sciences of Ukraine, 60 Nauky Ave., 61072 Kharkiv, Ukraine

## Abstract

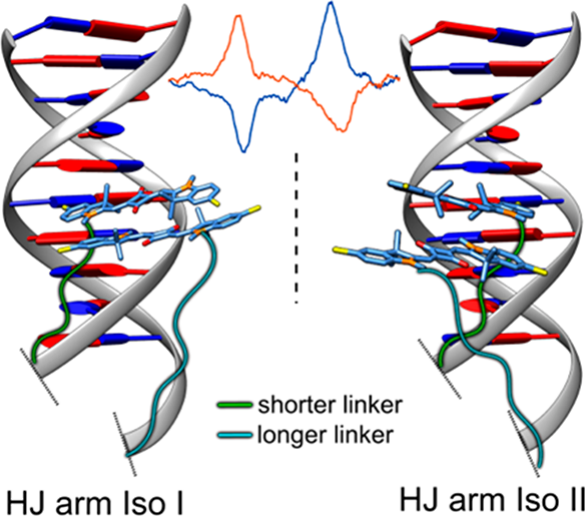

While only one enantiomer of chiral biomolecules performs a biological function, access to both enantiomers (or enantiomorphs) proved to be advantageous for technology. Using dye covalent attachment to a DNA Holliday junction (HJ), we created two pairs of dimers of bis(chloroindolenine)squaraine dye that enabled strongly coupled molecular excitons of opposite chirality in solution. The exciton chirality inversion was achieved by interchanging single covalent linkers of unequal length tethering the dyes of each dimer to the HJ core. Dimers in each pair exhibited profound exciton-coupled circular dichroism (CD) couplets of opposite signs. Dimer geometries, modeled by simultaneous fitting absorption and CD spectra, were related in each pair as nonsuperimposable and nearly exact mirror images. The origin of observed exciton chirality inversion was explained in the view of isomerization of the stacked Holliday junction. This study will open new opportunities for creating excitonic DNA-based materials that rely on programmable system chirality.

Because of the foundational role of molecular excitons (Frenkel excitons) in excitation energy transfer in natural photosynthetic antenna,^1,2^ they have been utilized in such applications as light harvesting,^3−5^ photonics,^6^ optoelectronics,^7^ organic photoswitches,^8−10^ and nanoscale computing.^11,12^ Molecular excitons are enabled by closely positioned dyes, i.e., dye aggregates, where excitonic coupling enables excitation to be shared between the dyes in a wavelike manner.^13^ Deoxyribonucleic acid (DNA) has been proven to be a versatile scaffold to organize dye molecules into dye aggregates. Its versatility stems from (1) the relative stability of DNA-templated dye aggregates and (2) the simplicity of design principles on the basis of the Watson–Crick pairing of four nucleotide building blocks.^14^ The covalent attachment of dyes to specific positions on the DNA via linkers allows control over the distance between the dyes as well as the number of dyes per aggregate.^5,6,15−31^ Moreover, dyes covalently templated by DNA typically adopt a distinct mutual orientation in their aggregate, which generally leads to a chiral configuration of the dyes’ constituent transition dipole moments observed in exciton-coupled circular dichroism (CD).^16,20,21,23,24,27,28,31,32^ In a simple dye aggregate of two dyes, i.e., a dimer, where dyes are attached to the neighboring nucleotides via short linkers, the excitons are typically right-handed^16,20,21,23−25,27,28,30−32^ likely because of the intrinsic chirality of the DNA helix. Left-handed excitons can also occur in specific types of dye attachment.^20,21,28^ However, there are no reported methods to create mirror image dye dimers (dimer enantiomorphs) that have inverted exciton chirality in solution. Such methods can lead to new functionalities of excitonic DNA-based materials and devices that rely on a strong, programmable, and sensitive-to-stimuli chiroptical response.^33^

In recent years, our group has used an immobile four-arm DNA scaffold, known as the Holliday junction (HJ),^34,35^ to create dye aggregates by covalently tethering dyes to the core of HJ. The HJ adopts an antiparallel stacked-X conformation in solutions containing divalent cations such as Mg^2+^ or high concentrations of monovalent cations such as Na^+^.^36−38^ In the stacked conformation, pairs of HJ arms are stacked with each other to form two coaxial continuous duplexes. The stacked HJ is known to exist in a dynamic equilibrium between two isomeric conformers, *Iso I* and *Iso II*, that differ in the pairs of stacked arms.^37^

We tethered dyes to HJ-constituent single strands via traditional short phosphoramidite linkers during the solid phase oligonucleotide synthesis,^32^ but also via single ∼2 nm long carbon linkers that offer the convenience of NHS-ester-type labeling as a postmodification of oligonucleotides.^39−43^ Since a long flexible linker allows for more motion of the dye, it might result in less control over the dye-specific orientation relative to the DNA, thereby reducing the capability to transfer the chirality of DNA to a dye aggregate. However, squaraine dyes tethered to thymidines in the core of the HJ via single, long linkers resulted in chiral excitons within the dimer^40,43^ or tetramer aggregates,^40^ as observed in their circular dichroism (CD) spectra. These results suggest that a distinct dye orientation might be induced by noncovalent binding between the dyes and a specific region of DNA. These aggregates exhibited medium-to-strong excitonic coupling, as defined in terms of excitonic hopping parameter *J*_*m,n*_.^13^ The configuration of dye attachment to the HJ core appeared to influence the strength of the CD couplets of squaraine dimers. Specifically, a transverse dimer, where dyes were attached to noncomplementary strands of an immobile HJ, exhibited a well-defined CD couplet, while the adjacent dimer, where the dyes were attached to partially complementary strands, exhibited a relatively weak CD signal.

In this work, we demonstrated a way to increase the intensity of the CD signal in the adjacent dimer and invert its exciton chirality in a controlled manner. The chirality inversion was achieved by mismatching the length of carbon linkers in a dye dimer that governed the conformation of the DNA HJ template. The resulting chiral exciton systems were associated with increased system homogeneity, which is critical for the fundamental studies of excitons and improved performance of exciton-based materials. This system also offers new opportunities for creating chiral DNA nanomaterials for use in DNA-based sensors and switches, data storage, and chemical synthesis and catalysis.

We created squaraine dimers and avoided homologous strand migration by employing an immobile HJ consisting of four strands, where each strand has partial complementarity to its two other adjacent neighbor strands and no complementarity to the other two strands. We chose a bis(chloroindolenine)squaraine dye that previously showed strong excitonic coupling in its dimers and tetramers when templated by HJ.^40^ The *N*-carboxypropyl, *N*-carboxypentyl, and *N*-carboxyheptyl derivatives of bis(chloroindolenine)squaraine were attached via amidation to amino C6 thymine modifiers placed in the centers of single DNA strands (Figure 1a, Table S1). The resulting dye-linker moieties are shown in the inset of Figure 1a, where linkers R_1_ (short), R_2_ (medium), and R_3_ (long) differ by two incremental methylene groups. The immobile HJs templating squaraine dimers were assembled by hybridizing equimolar amounts of two unlabeled single strands, A and C, and two dye-labeled single strands, SQ-B and SQ-C. The samples were annealed at 95 °C for 4 min followed by a slow 1.0 °C/min cooling to room temperature. In this manner, we created six dimers with unequal linker lengths (Figure 1b). For reference constructs, HJs templating three dimers with equal linker lengths and HJs templating a single dye via a short, medium, or long linker, i.e., monomers, were analogously synthesized (Figure 1c,d).

**Figure 1:**
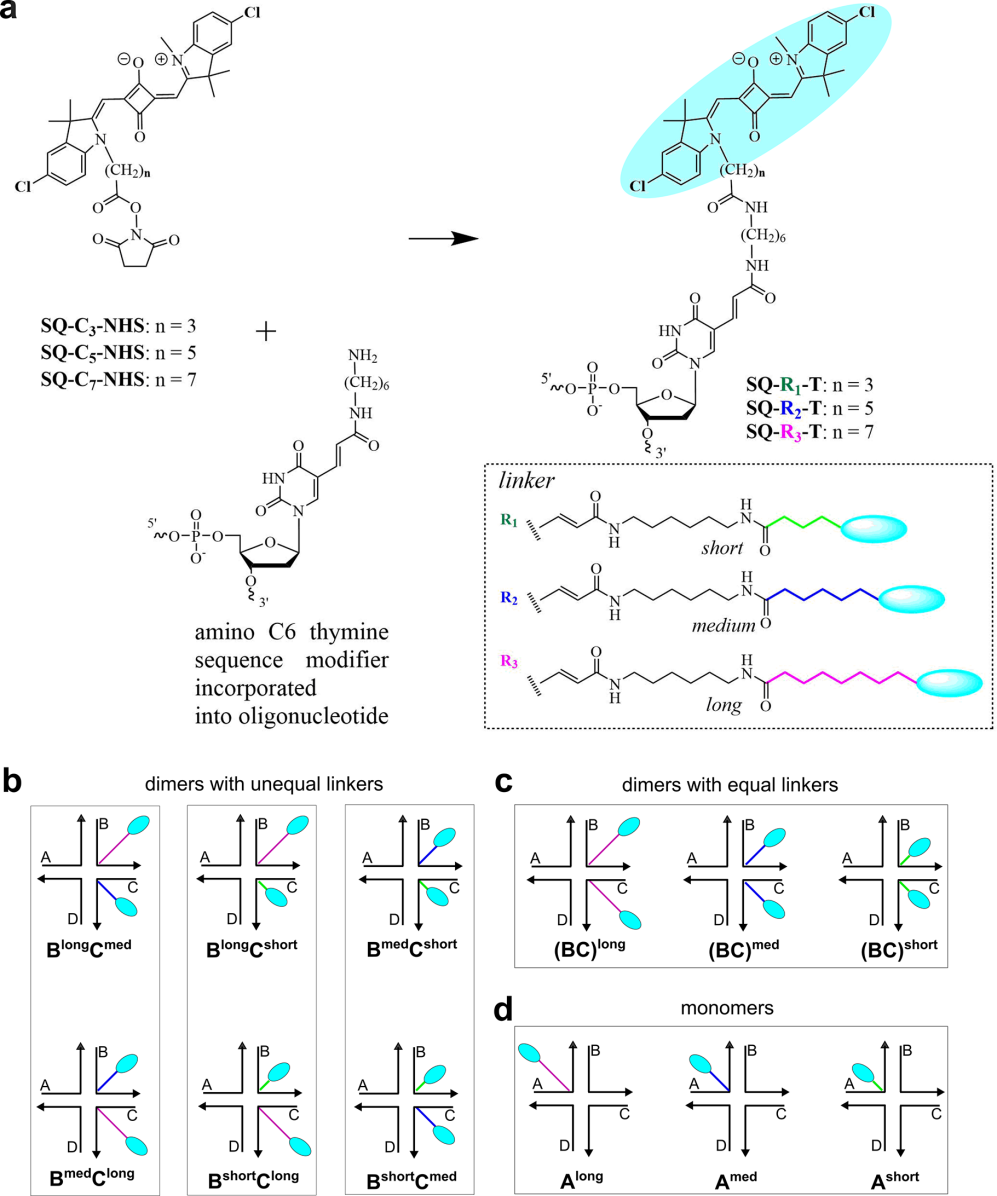
Molecular designs of squaraine–HJ constructs with three different linker lengths: short (green), medium (med; blue), and long (magenta). (a) General procedure for oligonucleotide postmodification with a squaraine dye depicting chemical structures of the resulting squaraine-labeled thymidines, linkers tethering squaraines to thymidines, and bis(chloroindolenine)squaraine. (b) Schematics of dimers with unequal linkers tethered to immobile HJ. (c) Schematics of dimers with equal linkers tethered to immobile HJ. (d) Schematics of monomers with varying linker lengths tethered to immobile HJ. The strands of immobile HJ are labeled A, B, C, and D.

The stabilities of dimer–DNA constructs were assessed by thermal denaturation and electrophoretic analysis (Sections S3 and S4). The DNA denaturation was monitored by absorption in the nucleobase region at 260 nm (Figure S6). The melting temperatures (*T*_m_) were determined from the maximum of the first derivative of the absorbance signal (dA/dT). Thermal denaturation of the control unlabeled HJ in 1× TBE, 15 mM MgCl_2_ produced a sigmoidal curve of one transition with *T*_m_ = 58.4 °C, which is characteristic of a stacked HJ conformation.^44^ The same HJ in an open conformation in 100 mM NaCl was previously observed to have a less cooperative unfolding and *T*_m_ = 46.7 °C.^41^ Melting profiles of the dimer–DNA constructs were very similar to that of the control HJ in MgCl_2_, which indicated that HJs templating squaraine dimers maintain the stacked conformation. Melting temperatures of dimers with equal and unequal linkers were in the range of 60.5–61.4 °C and, therefore, higher than the *T*_m_ of unlabeled HJ. These results are indicative of dye–dye interactions having an overall stabilizing effect on the dimer–DNA constructs containing unpaired thymine modifiers in the junction core. The stacked HJ conformation of the dimer–DNA constructs was further supported by nondenaturing polyacrylamide gel electrophoresis carried out in 1× TBE, 15 mM MgCl_2_ running buffer. All dimer–DNA constructs exhibited well-defined bands of the same electrophoretic mobility as the unlabeled HJ. Only negligible amounts of ssDNA and higher order constructs were observed in a few samples (Figure S7, Section S4).

We characterized the squaraine monomers and dimers with steady-state absorption and circular dichroism spectroscopies (Figure 2). The monomers exhibited characteristic spectral features of squaraines, including an intense absorption spectral band with a peak maximum at 645 nm accompanied by a small vibronic shoulder (Figure 2a). The monomers did not produce a signal in the visible region of the CD spectrum, which indicated that DNA HJ does not induce chirality of achiral squaraines (Figure 2b). Absorption is useful for observing dye aggregation and qualitative estimation of excitonic coupling. Regardless of linker length, all dimers exhibited very similar absorption profiles with the high-energy absorption band at 596 nm. These absorption profiles corresponded to a blue shift and an increase in the intensity of the monomer’s absorption (Figure 2c,e,g,i). According to Kasha’s excitonic model,^13^ such spectral changes indicated the formation of a dimer aggregate with face-to-face dye orientation (H-aggregate) and strong excitonic coupling.

**Figure 2:**
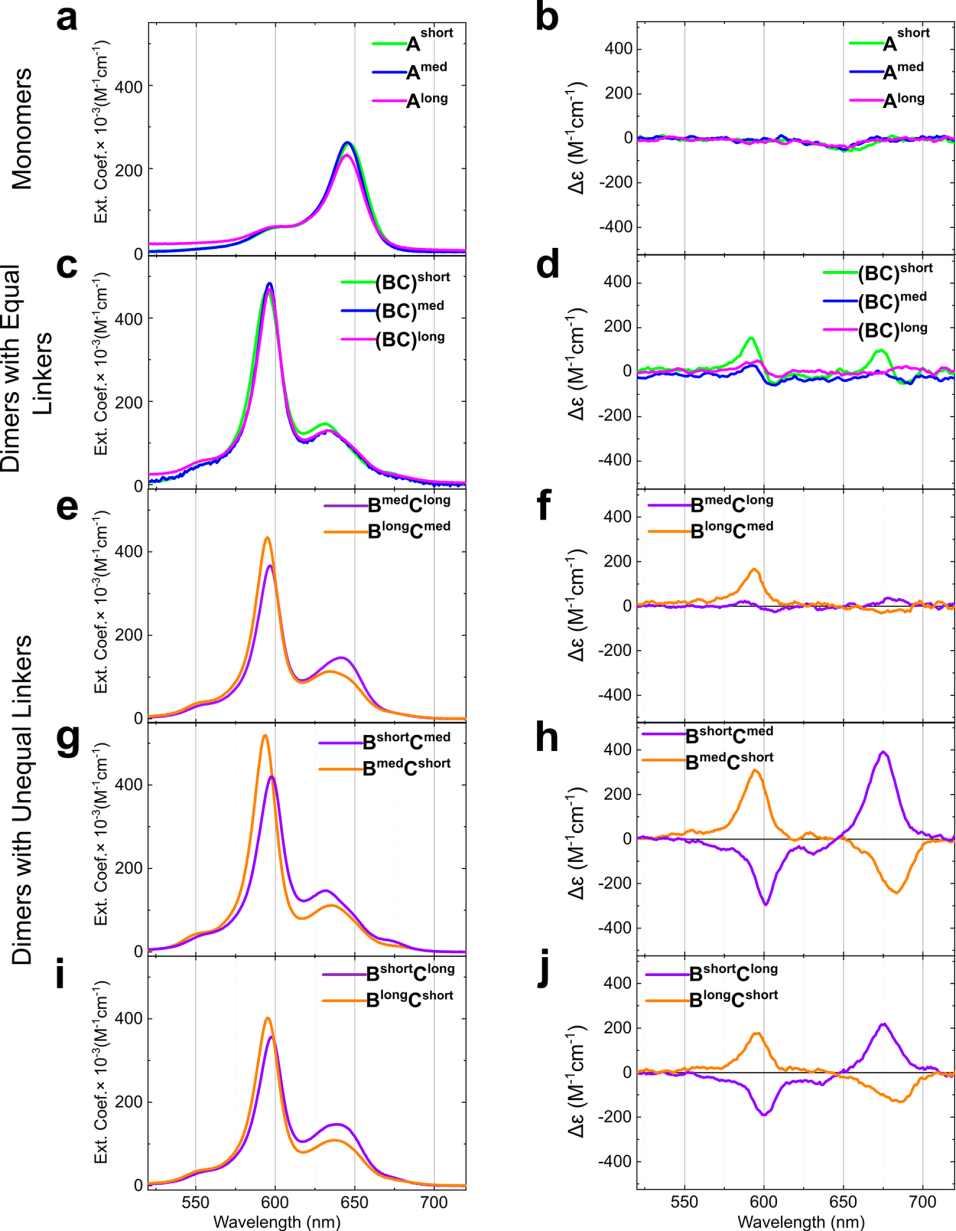
Steady-state absorption and circular dichroism of squaraine monomers and dimers tethered to HJ. The spectra were recorded in 1× TBE, 15 mM MgCl_2_ at room temperature. The concentrations of squaraine–DNA constructs were 1.5 μM. (a) Absorption profiles of single bis(chloroindolenine)squaraine covalently attached to strand A of HJ (i.e., monomers) do not show dependence on the length of the linker. (b) CD spectra of achiral bis(chloroindolenine)squaraine covalently attached to strand A of HJ (i.e., monomers) do not exhibit an induced CD signal. (c) Absorption profiles of dimers with equal linkers are blue-shifted relative to the monomers, indicative of the presence of excitonic coupling. (d) CD profiles of dimers with equal linkers show exciton-coupled CD signals with low amplitude. (e,g,i) Absorption profiles of dimers with unequal linkers are blue-shifted relative to the monomers, indicative of the presence of excitonic coupling. (f) CD profile of dimers **B**^**med**^**C**^**long**^ and **B**^**long**^**C**^**med**^ show exciton-coupled CD signal with low magnitude. (h,j) CD profiles of dimer pairs **B**^**long**^**C**^**short**^/**B**^**short**^**C**^**long**^ and **B**^**med**^**C**^**short**^/**B**^**short**^**C**^**med**^ show high-magnitude exciton-coupled CD signals of opposite handedness.

CD measurements are useful for observing exciton chirality.^45^ While all examined dimers showed signs of dimer formation with strong excitonic coupling in the absorption spectra, only two pairs of dimers with unequal linker length, namely **B**^**med**^**C**^**short**^/**B**^**short**^**C**^**med**^ and **B**^**long**^**C**^**short**^/**B**^**short**^**C**^**long**^, exhibited pronounced bisignate couplets with large magnitudes in their CD spectra (Figure 2h,j). Since the squaraine monomers do not exhibit a CD signal in the visible region, we attributed the CD signals exhibited by the dimers to the exciton-coupled transition dipole moments (TDMs). The Cotton effects showed similar peak maxima and minima, though the magnitude of the peak for the short–long linker combination was smaller than for the medium–short linker combination. Moreover, interchanging the linkers in each construct (i.e., **B**^**med**^**C**^**short**^ vs **B**^**short**^**C**^**med**^ and **B**^**long**^**C**^**short**^ vs **B**^**short**^**C**^**long**^) resulted in inverted CD couplets. Constructs with a shorter linker on strand B were characterized by a positive Cotton effect (up–down shape from right to left) and were described as right-handed (i.e., have positive chirality). In contrast, constructs with a shorter linker on strand C were characterized by a negative Cotton effect (down–up shape from right to left), and we described them as left-handed (i.e., have negative chirality). While the same behavior in the CD spectra was observed for dimers with a medium–long linker combination, i.e., **B**^**med**^**C**^**long**^ and **B**^**long**^**C**^**med**^, the magnitudes of the CD couplets were very weak in these constructs (Figure 2f). We ensured that the dimers exhibiting exciton chirality were thermodynamically stable structures by reannealing their samples under the initial annealing conditions after nine months of storage. The refolded dimer constructs exhibited the same handedness as the initially prepared dimers (Figure S10). The fact that dimer handedness was preserved upon refolding indicated that dimer formation (i.e., dye alignment within a dimer) and DNA folding is a cooperative event where dimer chirality is predetermined by the thermodynamically stable structure of the HJ templating the dimer.

The reference dimers with equal linker lengths **(BC)**^**short**^, **(BC)**^**med**^, and **(BC)**^**long**^, were characterized by an absent to a very weak CD signal in the visible region. However, the absence of a CD signal does not mean there are no excitonically coupled TDMs. This absence may be attributable to the following: (1) several populations of excitonically coupled TDMs, i.e., a heterogeneous mixture; (2) an aggregate having an internal symmetry in the orientation of TDMs (TDMs are aligned strictly in line or in parallel); or (3) a racemic mixture of two aggregates that are nonsuperimposable mirror images of each other (a racemate of enantiomorphs). We tested whether the third case, a racemic mixture, might be taking place in the constructs that had equal linker lengths by incrementally adding **B**^**long**^**C**^**short**^ to **B**^**short**^**C**^**long**^ while recording the changes in the CD profile of the resulting sample (Figure 3a). The CD signal of the sample with a 10:10 molar ratio of **B**^**short**^**C**^**long**^ and **B**^**long**^**C**^**short**^ nearly vanished, and its profile closely resembled the CD profile of **(BC)**^**med**^ dimer (Figure 2d). Similarly, the incremental addition of **B**^**short**^**C**^**med**^ to **B**^**med**^**C**^**short**^ (Figure 3b) resulted in a CD profile resembling that of the **(BC)**^**short**^ dimer (Figure 2d). These results indicated that the CD of the **(BC)**^**short**^ and **(BC)**^**med**^ dimers having equal linkers can result from the presence of two equimolar populations of dimers exhibiting opposite CD couplets, thus supporting the hypothesis that these dimers exist as nearly equimolar mixtures of enantiomorphs.

**Figure 3:**
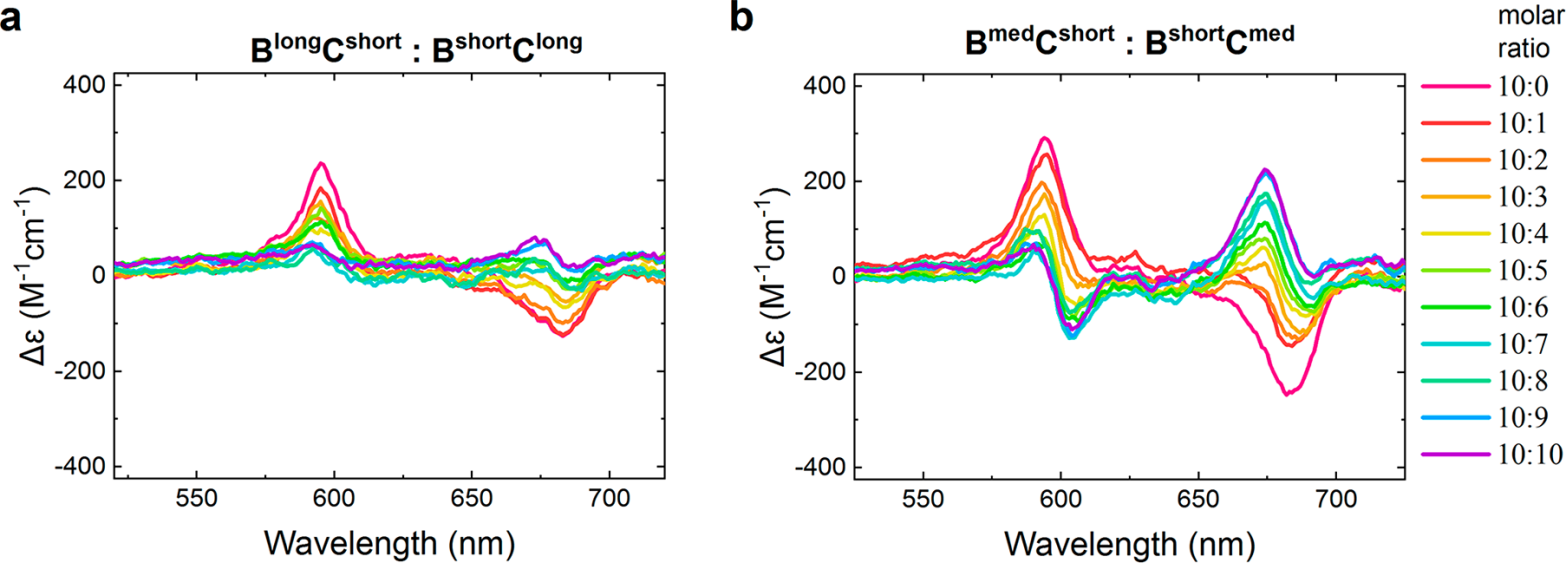
Changes in molar CD profiles of squaraine dimers upon incremental addition of the paired dimer with interchanged linkers._._ (a) Stepwise addition of dimer **B**^**short**^**C**^**long**^ [10× (2 μM, 10 μL)] to dimer **B**^**long**^**C**^**short**^ (2 μM, 100 μL) results in the CD profile similar to that of **(BC)**^**med**^. (b) Stepwise addition of dimer **B**^**short**^**C**^**med**^ [10× (2 μM, 10 μL)] to dimer **B**^**med**^**C**^**short**^ (2 μM, 100 μL) results in the CD profile similar to that of **(BC)**^**short**^. All measurements were performed in 1× TBE, 15 mM MgCl_2_ at room temperature.

We explained the chirality inversion in squaraine dimers with unequal linker length by considering the isomeric behavior of the stacked HJ. In one stacking isomer (arbitrary *Iso I*), strands A and C adopt continuous helical structures, while the other two strands, B and D, cross over at the branch point. In the second stacking isomer (then *Iso II*), the strand folding is reversed (Figure 4a). While the global structures of *Iso I* and *Iso II* are identical, within the global structure, the single strand intertwining in each arm relative to the branch point is opposite between *Iso I* and *Iso II*, according to the conventional isomerization model.^36,46^ The opposite configurations of the DNA strand intertwining between *Iso I* and *Iso II* result in the opposite base pairing in each HJ arm. Our thermal denaturation experiments showed that, in a buffer supplemented with 15 mM MgCl_2_, our dye-modified HJ constructs adopt the stacked conformation. As such, the relative positions of the dye-labeled thymidines in the given adjacent dimer configuration should remain the same in both *Iso I* and *Iso II* isomers and perhaps do not strongly influence the equilibrium between *Iso I* and *Iso II* (Figure 4b). With the assumption that a distinct dimer alignment is promoted by the noncovalent binding of a dimer’s one or both dyes to a specific region of the HJ arm, the geometry of the dye dimer in *Iso I* should be similar to the dye dimer in *Iso II*. The difference between the two dye dimers in *Iso I* and *Iso II* would be their handedness as the result of noncovalent binding to an opposite base pairing (Figure 4c).

**Figure 4:**
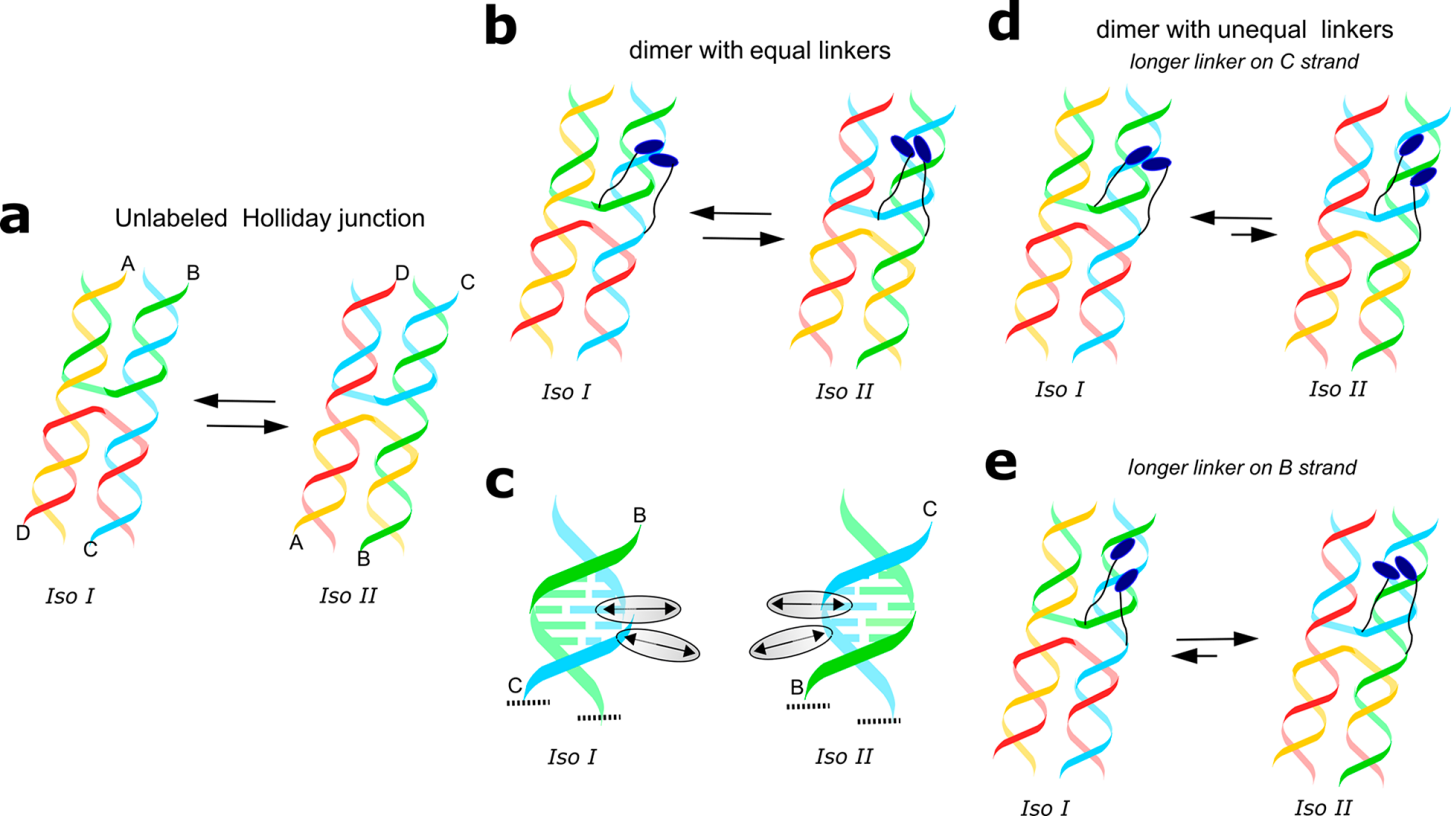
Schematic representation of the influence of linker length on an equilibrium between isomers of the stacked HJ covalently templating squaraine dimers. The transitional open conformation of HJ is not shown. (a) Unlabeled HJ at the dynamic equilibrium between *Iso I* and *Iso II* isomers. (b) *Iso I* and *Iso II* are equally favorable when dyes are covalently tethered to the HJ via equal-length linkers resulting in a racemic mixture of dimer enantiomorphs. (c) Noncovalent binding of dye dimer to the HJ arm in *Iso I* and *Iso II* isomers that feature opposite base pairing. (d,e) A shift in equilibrium toward a dominant HJ isomer when dyes are covalently tethered to the HJ via unequal-length linkers. A dominant isomer is the isomer where a dye–dye distance is shorter [i.e., *Iso I* in (d) and *Iso II* in (e)].

If *Iso I* and *Iso II* are equally favorable (1:1 ratio), which we assume is the case for dimers with equal linker lengths and for dimers **B**^**long**^**C**^**med**^/**B**^**med**^**C**^**long**^, two dimer enantiomorphs form a racemic mixture with each CD signal canceling the other. Alternatively, if one HJ isomer is dominant, then the CD spectrum corresponding to the enantiomorphic excess is observed. In the unlabeled HJs, a dominant isomer is known to be base-pair-sequence dependent. The base-pair sequence of the HJ core has been shown to have the most influence on the HJ isomer bias,^47−49^ with base pairs further from the core also affecting the isomer equilibrium in some cases.^50,51^ In contrast, our previous studies indicated that for a stacked HJ covalently templating a dye dimer, strongly attractive dye–dye interactions can make the isomer with a shorter dye–dye distance more dominant.^41^ If such interactions could be achieved only in one HJ isomer, the equilibrium would be shifted toward that isomer (*Iso I* in Figure 4d and *Iso II* in Figure 4e). In this study, an unequal linker length can prevent a mirror image dye alignment in one HJ isomer (e.g., where a shorter linker is attached to the dye-labeled thymidine at the farther end), thus favoring another HJ isomer with the dye alignment enabling stronger dye–dye interactions, which we consider to be a case for dimers **B**^**med**^**C**^**short**^/**B**^**short**^**C**^**med**^ and **B**^**long**^**C**^**short**^/**B**^**short**^**C**^**long**^ (Figure 4de). We tested the versatility of the given molecular design to promote exciton chirality inversion by examining three other series of adjacent dimers, namely, AD, AB, and DC (Figure S5), with unequal linkers prepared in the same manner as the BC dimers. The dimers AD, AB, and DC demonstrated the same exciton chirality inversion upon interchanging linkers of unequal length (combinations short–medium and short–long) as the BC dimers and did not show this effect with linker combination medium–long (Figures S11, S12, and S13). These results further support a strong influence of dye–dye interactions on the isomerization of the stacked HJ.

Last, we evaluated the excitonic coupling strength and geometries of dimers **B**^**short**^**C**^**long**^/**B**^**lo**ng^**C**^**short**^ and **B**^**med**^**C**^**short**^/**B**^**short**^**C**^**med**^ that enabled excitons of opposite chirality. We obtained these parameters by simultaneously fitting dimer absorption and CD spectra using the approach previously developed in our group^32,40,41^ on the basis of the Kühn-Renger-May (KRM) model (Section S6).^52^ We built the dimer geometries on the basis of the TDM orientations extracted from the KRM modeling. The geometry of each dimer was characterized by a center-to-center distance, *R*; slip angle, θ, to describe the sliding of one dye relative to another; and oblique angle, α (Figure 5). In general, the dimer pairs with interchanged linkers related as nonsuperimposable entities with a slight deviation from exact mirror images. The dimer pair **B**^**short**^**C**^**long**^/**B**^**lo**ng^**C**^**short**^ was the closest to an exact mirror image relationship. Interestingly, dimers with a longer linker on strand B had a shorter center-to-center distance and smaller obliquity than those with a shorter linker on strand B. All four bis(chloroindolenine)squaraine dimers exhibited excitonic coupling strength described by the excitonic hopping parameter *J*_1,2_ in the range of 97–121 meV, which corresponds to the strong coupling regime. Overall, the excitonic coupling in the bis(chloroindolenine)squaraine dimers with unequal linkers was only slightly smaller than that in previously characterized **(BC)**^**med**^ with equal medium length linkers (*J*_1,2_ = 132 meV)^40^ and stronger than in dimers of indolenine squaraines with other substituents.^40^

**Figure 5:**
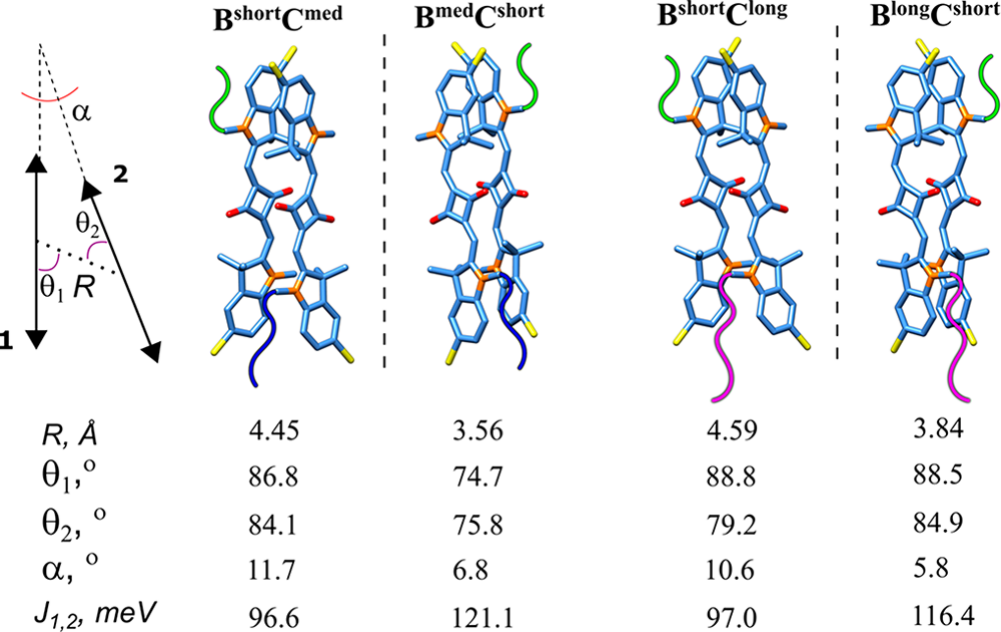
KRM-derived dimer geometries with unequal length linkers are shown in pairs as mirror images: **B**^**short**^**C**^**med**^/**C**^**med**^**B**^**short**^ and **B**^**short**^**C**^**long**^/**B**^**lo**ng^**C**^**short**^. The left schematic defines the geometric parameters of the alignment between TDMs 1 and 2 in each dimer: a center-to-center distance *R* in Å, slip angles θ_1_*°* and θ_2_*°*, and an oblique angle α*°*. Note that the fitting procedure determines the position and orientation of TDM that aligns with the long axes of the squaraine dye but not the rotation of the dye core around its long axis. As such, the dye core rotations were arbitrarily chosen.

On the basis of the CD experiments toward a racemic mixture (Figure 3), we can treat the geometries of the dimers obtained for biased *Iso I* and *Iso II* isomers as the geometries of the dimers in equilibrium when there is no conformational bias. In particular, the geometries of dimers **B**^**short**^**C**^**long**^ and **B**^**long**^**C**^**short**^ can be considered the geometries of the dimer **(BC)**^**med**^ in equilibrium between *Iso I* and *Iso II*. The modeling results also show that while the geometric parameters of the dimers **B**^**short**^**C**^**long**^ and **B**^**long**^**C**^**short**^ are very similar, they are not identical. This means that **(BC)**^**med**^ dimer with equal linkers (or any other aggregate that does not impose a conformational bias on the stacked HJ isomerization) may exist as a population of two similar dimers contributing to the system heterogeneity. In contrast, unequal linkers tethering a dimer to the HJ core afford one dimer population and, therefore, a more homogeneous system.

In conclusion, we demonstrated a system that uses a dye dimer covalently templated by HJ, where the exciton chirality can be programmed to be right-handed or left-handed. In terms of organization and scale, our system is comparable to the systems for DNA-mediated asymmetric catalysis. In DNA constructs for asymmetric catalysis, small molecule ligands covalently attached to dsDNA form a catalytic site whose chirality can be inverted by changing the DNA conformation from right-handed helix to left-handed helix.^53−55^ In contrast, here we discussed an opposite mechanism of chirality inversion in which the dynamic conformation of DNA is governed by the chemical structure of the ligands attached to it, and the exciton chirality is enabled by opposite base pairing within the right-handed helix. We believe this study opens a wide range of new opportunities for both fundamental studies of HJ and the creation of DNA-based materials (excitonic and beyond), such as sensors and switches, data storage, and DNA-mediated chemical synthesis and catalysis.

## Experimental Methods

### Sample Preparation

We purchased DNA oligomers from Integrated DNA Technologies (IDT) and specified that they were internally functionalized with custom squaraines via amino C6 thymine sequence modifiers and purified via dual high-performance liquid chromatography. Nonfunctionalized DNA oligomers purified by standard desalting were also purchased from IDT. All DNA oligomers were rehydrated in ultrapure water (Barnstead Nanopure, Thermo Scientific) to prepare 100 μM stock solutions. Concentrations of DNA samples were verified spectroscopically on a NanoDrop One Microvolume UV–Vis Spectrophotometer (Thermo Scientific) using calculated extinction coefficients. DNA Holliday junctions were prepared by combining equimolar amounts of four oligomers in 1× TBE, 15 mM MgCl_2_ buffer solution to a final DNA concentration of 1.5 μM for steady-state absorption and CD measurements and 2.0 μM for stepwise CD measurements of dimer mixtures. All DNA samples were annealed in a Mastercycler Nexus PCR cycler (Eppendorf) according to the following protocol: 4 min at 95 °C, followed by cooling at 1.0 °C/min from 95 to 25 °C.

### Optical Characterization

UV–vis spectra were recorded in duplicates at room temperature on a dual-beam Cary 5000 UV–vis–NIR spectrophotometer (Agilent Technologies) in a quartz cuvette with a 1 cm path length (Starna). Absorption spectra were monitored over a 230–800 nm wavelength range. CD measurements were performed on a J-1500 spectropolarimeter (Jasco). DNA samples (120 μL) were transferred to a 1 cm path-length quartz cuvette. Spectra were recorded over the 230–800 nm wavelength range at a speed of 200 nm min^–1^ (three scans per sample were averaged).

## Supporting Information Available

Chemical synthesis of squaraine dyes; oligonucleotide sequences; thermal denaturation; electrophoretic analysis; full region absorption and CD spectra of BC, AD, AB, and DC dimers; and KRM modeling, including dimers **B**^**short**^**C**^**med**^ and **B**^**med**^**C**^**short**^ (PDF)
