# Supplementary material for: Exciton Chirality Inversion in Dye Dimers Templated by DNA Holliday Junction
Source: J Phys Chem Lett. 2022 Nov 10;13(46):10688–96. doi: 10.1021/acs.jpclett.2c02721 (PMC9706552; doi:10.1021/acs.jpclett.2c02721)
Supplement: Supplementary file 1 — jz2c02721_si_001.pdf [file jz2c02721_si_001.pdf]

SUPPORTING INFORMATION

Exciton Chirality Inversion in Dye Dimers Templated by DNA Holliday Junction

Olga A. Mass,[1,*] Shibani Basu,[1] Lance K. Patten,[1] Ewald A. Terpetschnig,[2] Alexander I. Krivoshey,[3] Anatoliy L. Tatarets,[3] Ryan D. Pensack,[1] Bernard Yurke,[1,4] William B. Knowlton, [1,4] and Jeunghoon Lee[1,5,*]

[1]Micron School of Materials Science & Engineering, [4]Department of Electrical & Computer Engineering, [5]Department of Chemistry and Biochemistry, Boise State University, Boise, Idaho 83725, United States

[2]SETA BioMedicals, LLC, 2014 Silver Court East, Urbana, IL 61801, United States

[3]SSI "Institute for Single Crystals" of the National Academy of Sciences of Ukraine, 60 Nauky Ave., 61072 Kharkiv, Ukraine

Corresponding authors:

*olgamass@boisestate.edu
*jeunghoonlee@boisestate.edu

Table of Contents

SI1	Squaraine Chemical Synthesis	S2
SI2	Oligo Sequences	S9
SI3	Thermal Denaturation	S11
SI4	Electrophoretic Analysis	S13
SI5	Full Absorption and CD Spectra of Squaraine Monomers and Dimers	S15
SI6	Fitting Absorption and CD using KRM Model	S21
SI7	References	S32

Supporting Information 1: *Squaraine Chemical Synthesis*

General information

The **C, H, and N elemental analysis** was performed by a EuroVector Euro EA 3000 EA-IRMS elemental analyzer.

^{1}H NMR spectra were measured on a Varian 400 MR (^{1}H 400 MHz) spectrometer in DMSO-d_6 using the signal of the remaining non-deuterated solvent as an internal standard (2.50 ppm for DMSO[1]).

ESI mass spectra were recorded on *Waters Quattro micro API* mass spectrometer with direct injection of the sample solution into the ionization chamber. Spectra were recorded in ESI$^+$ at 120 °C with energy 3 kV on the capillary.

The purity of the obtained compounds was monitored by **HPLC** on Agilent Technologies 1100 (LC), column Phenomenex Luna Omega 5 µm C18 100 Å, 4.6 × 250 mm, column temperature 35 °C, eluent water-acetonitrile (ACN) + 0.05% phosphoric acid.

Absorption spectra were recorded in 1-cm quartz cells at 25 ºC using a PerkinElmer Lambda 35 UV/Vis spectrophotometer. Absorption maxima were determined with an accuracy of ±0.5 nm and rounded off.

Synthesis

8-Bromooctanoic acid was from Synthonix; all other reagents and Silica gel 60 for column chromatography were from Aldrich and used as received. 3-((5-Chloro-1,3,3-trimethylindolin-2-ylidene)methyl)-4-hydroxycyclobut-3-ene-1,2-dione was synthesized according to procedure.[2] Compound **SQ-C5-NHS** was synthesized as described in the literature.[3]

2-((1-(3-carboxypropyl)-5-chloro-3,3-dimethylindolin-2-ylidene)methyl)-4-((5-chloro-1,3,3-trimethyl-3*H*-indol-1-ium-2-yl)methylene)-3-oxocyclobut-1-en-1-olate (**SQ-C3-COOH**).

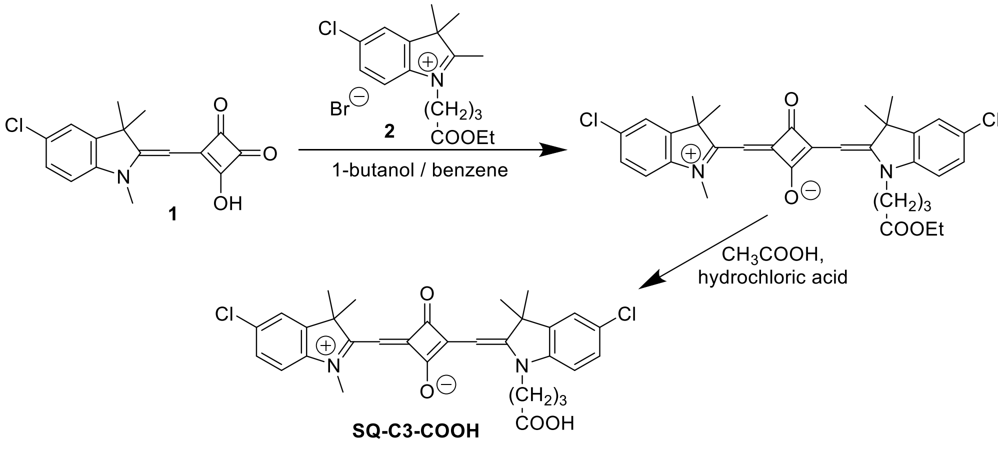

3-((5-Chloro-1,3,3-trimethylindolin-2-ylidene)methyl)-4-hydroxycyclobut-3-ene-1,2-dione (**1**) (200 mg, 0.66 mmol) and 5-chloro-1-(4-ethoxy-4-oxobutyl)-2,3,3-trimethyl-3*H*-indol-1-ium bromide (**2**) (256 mg, 0.66 mmol) were heated under reflux in 1-butanol (25 mL) and benzene (5 mL) using a Dean-Stark trap for 20 h. The solvent was removed under reduced pressure by a rotary evaporator. The residue containing esterified dye was hydrolyzed in a mixture of acetic acid (25 mL) and concentrated hydrochloric acid (2.5 mL) at 100 °C for 1 h. Then acids were removed under reduced pressure by a rotary evaporator, and raw product was purified by column

chromatography (Silica gel 60, 2–6% methanol—chloroform) to give **SQ-C3-COOH** (165 mg, 44%). ^{1}H-NMR (400 MHz, DMSO-d$_6$), δ, ppm: 7.65 (2H, t, arom., 2.3 Hz), 7.43–7.31 (4H, m, arom.), 5.85 (1H, s, C*H*), 5.77 (1H, s, C*H*), 4.09 (2H, t, NCH_2), 3.56 (3H, s, NCH_3), 2.37 (2H, t, CH_2COOH, 7.1 Hz), 1.93–1.80 (2H, m, CH_2), 1.68 (12H, s, (CH_3)$_2$). ESI MS, m/z calcd. for $[M+H]^+$ $[C_{31}H_{31}Cl_2N_2O_4]^+$ 565.17, found: 565.21. Anal. calcd. (%) for $C_{31}H_{30}Cl_2N_2O_4$: C, 65.84; H, 5.35; N, 4.95. Found C, 65.99; H, 5.43; N, 4.93. UV-Vis: λ_{max}(Abs) 633 nm, ε 250,000 $M^{-1}cm^{-1}$ (Methanol); λ_{max}(Em) 644 nm (Methanol).

4-((5-chloro-1,3,3-trimethyl-3*H*-indol-1-ium-2-yl)methylene)-2-((5-chloro-1-(4-((2,5-dioxopyrrolidin-1-yl)oxy)-4-oxobutyl)-3,3-dimethylindolin-2-ylidene)methyl)-3-oxocyclobut-1-en-1-olate (*N*-Hydroxysuccinimide ester of **SQ-C3-COOH**) (**SQ-C3-NHS**).

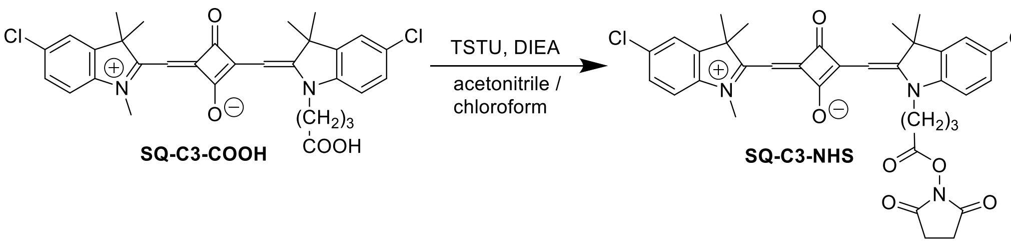

SQ-C3-COOH (42.7 mg, 75.5 μmol), *N*,*N*,*N'*,*N'*-tetramethyl-*O*-(*N*-succinimidyl)uronium tetrafluoroborate (TSTU) (30 mg, 99.6 μmol) were suspended in acetonitrile (5 mL) and chloroform (0.5 mL), and then *N*,*N*-diisopropylethylamine (DIEA) (20 μL, 114.8 μmol) was added. The solution was stirred at room temperature for 30 min. The solvent was removed under reduced pressure by a rotary evaporator. The residue was purified by column chromatography (Silica gel 60, 0.5–2% methanol—chloroform) to give **SQ-C3-NHS**. Yield: 25 mg (50%). ^{1}H-NMR (400 MHz, DMSO-d$_6$), δ, ppm: 7.68 (1H, s, arom.), 7.66 (1H, s, arom.), 7.45–7.32 (4H, m, arom.), 5.86 (1H, s, C*H*), 5.78 (1H, s, C*H*), 4.15 (2H, t, NCH_2), 3.57 (3H, s, NCH_3), 2.95 (2H, t, CH_2COONHS, 7.0 Hz), 2.86 (4H, s, CH_2 (succinimide)), 2.06–1.96 (2H, m, CH_2), 1.68 (12H, s, (CH_3)$_2$). ESI MS, m/z calcd. for $[M+H]^+$ $[C_{35}H_{34}Cl_2N_3O_6]^+$ 662.18, found: 662.21. Anal. calcd. (%) for $C_{35}H_{33}Cl_2N_3O_6$: C, 63.45; H, 5.02; N, 6.34. Found C, 63.54; H, 5.07; N, 6.26. UV-Vis: λ_{max}(Abs) 633 nm; λ_{max}(Em) 644 nm (Methanol).

2-((1-(7-carboxyheptyl)-5-chloro-3,3-dimethylindolin-2-ylidene)methyl)-4-((5-chloro-1,3,3-trimethyl-3*H*-indol-1-ium-2-yl)methylene)-3-oxocyclobut-1-en-1-olate (SQ-C7-COOH).

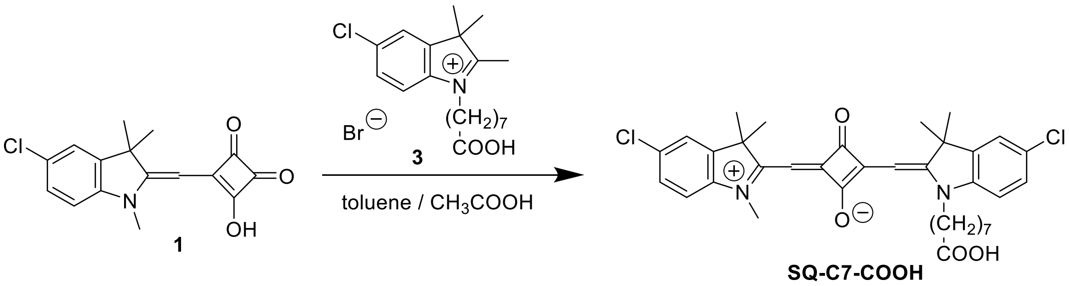

3-((5-Chloro-1,3,3-trimethylindolin-2-ylidene)methyl)-4-hydroxycyclobut-3-ene-1,2-dione (**1**) (200 mg, 0.66 mmol) and 1-(7-carboxyheptyl)-5-chloro-2,3,3-trimethyl-3*H*-indol-1-ium bromide (**3**) (274 mg, 0.66 mmol) were heated under reflux in toluene (25 mL) and acetic acid (5 mL) using

a Dean-Stark trap for 20 h. The solvent was removed under reduced pressure by a rotary evaporator. The residue was purified by column chromatography (Silica gel 60, 0–3% methanol—chloroform) to give **SQ-C7-COOH** (162 mg, 40%). ^{1}H-NMR (400 MHz, DMSO-d$_6$), δ, ppm: 7.65 (2H, s, arom.), 7.44–7.29 (4H, m, arom.), 5.79 (1H, s, C*H*), 5.76 (1H, s, C*H*), 4.06 (2H, broad s, NC*H*$_2$), 3.56 (3H, s, NC*H*$_3$), 2.18 (2H, t, C*H*$_2$COOH, 6.8 Hz), 1.68 (14H, s, (C*H*$_3$)$_2$ + C*H*$_2$), 1.54–1.42 (2H, m, C*H*$_2$), 1.41–1.20 (6H, m, C*H*$_2$). ESI MS, m/z calcd. for $[M+H]^+$ $[C_{35}H_{39}Cl_2N_2O_4]^+$ 621.23, found: 621.25. Anal. calcd. (%) for $C_{35}H_{38}Cl_2N_2O_4$: C, 67.63; H, 6.16; N, 4.51. Found C, 67.74; H, 6.19; N, 4.48. UV-Vis: λ_{max}(Abs) 634 nm, ε 256,000 $M^{-1}cm^{-1}$ (Methanol); λ_{max}(Em) 644 nm (Methanol).

4-((5-chloro-1,3,3-trimethyl-3*H*-indol-1-ium-2-yl)methylene)-2-((5-chloro-1-(8-((2,5-dioxopyrrolidin-1-yl)oxy)-8-oxooctyl)-3,3-dimethylindolin-2-ylidene)methyl)-3-oxocyclobut-1-en-1-olate (*N*-Hydroxysuccinimide ester of **SQ-C7-COOH**) (**SQ-C7-NHS**).

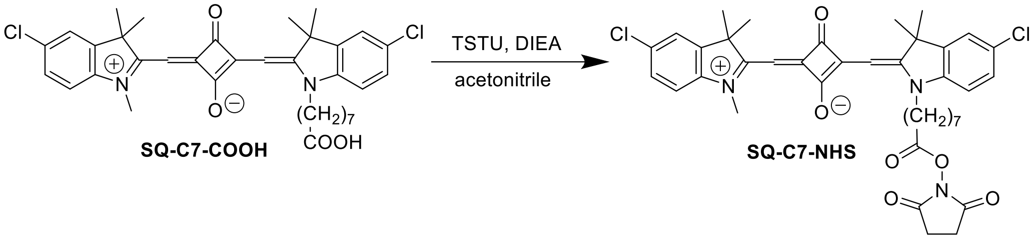

SQ-C7-COOH (43.0 mg, 69.2 μ mol), *N*,*N*,*N'*,*N'*-tetramethyl-*O*-(*N*-succinimidyl)uronium tetrafluoroborate (TSTU) (35 mg, 116.2 μmol) were suspended in acetonitrile (5 mL), and then *N*,*N*-diisopropylethylamine (DIEA) (24 μL, 137.7 μmol) was added. The solution was stirred at room temperature for 20 min. The solvent was removed under reduced pressure by a rotary evaporator. The residue was purified by column chromatography (Silica gel 60, 0–2% methanol—chloroform) to give **SQ-C7-NHS**. Yield: 37 mg (74%). ^{1}H-NMR (400 MHz, DMSO-d$_6$), δ, ppm: 7.65 (2H, s, arom.), 7.43–7.28 (4H, m, arom.), 5.80 (1H, s, C*H*), 5.76 (1H, s, C*H*), 4.06 (2H, broad s, NC*H*$_2$), 3.55 (3H, s, NC*H*$_3$), 2.80 (4H, s, CH$_2$ (succinimide)), 2.65 (2H, C*H*$_2$COONHS), 1.68 (12H, s, (C*H*$_3$)$_2$) , 1.63–1.57 (2H, m, C*H*$_2$), 1.43–1.28 (6H, m, C*H*$_2$), 1.28–1.18 (2H, m, C*H*$_2$). ESI MS, m/z calcd. for $[M+H]^+$ $[C_{39}H_{42}Cl_2N_3O_6]^+$ 718.24, found: 718.26. Anal. calcd. (%) for $C_{39}H_{41}Cl_2N_3O_6$: C, 65.18; H, 5.75; N, 5.85. Found C, 65.33; H, 5.79; N, 5.73. UV-Vis: λ_{max}(Abs) 634 nm; λ_{max}(Em) 644 nm (Methanol).

^{1}H NMR spectra of the obtained compounds

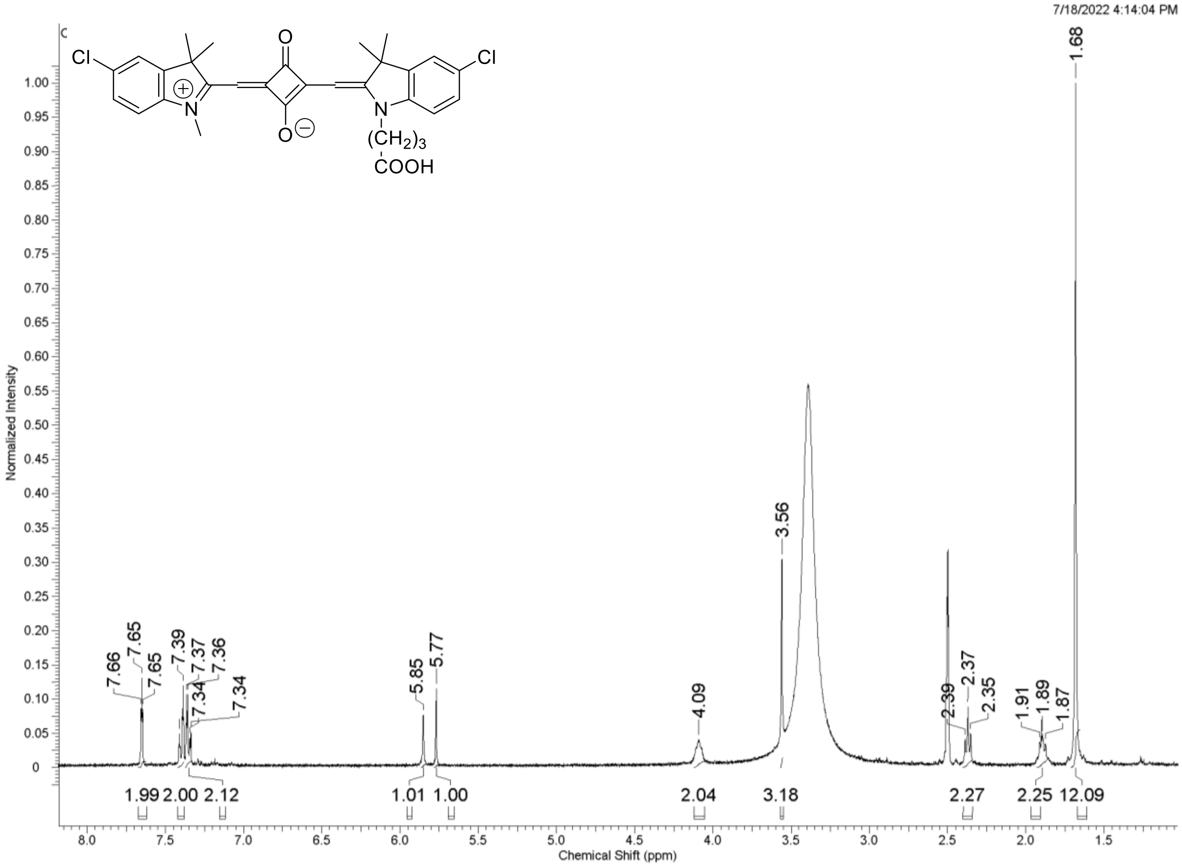

Figure S1. ^{1}H NMR spectrum of **SQ-C3-COOH** (400 MHz, DMSO-d_6).

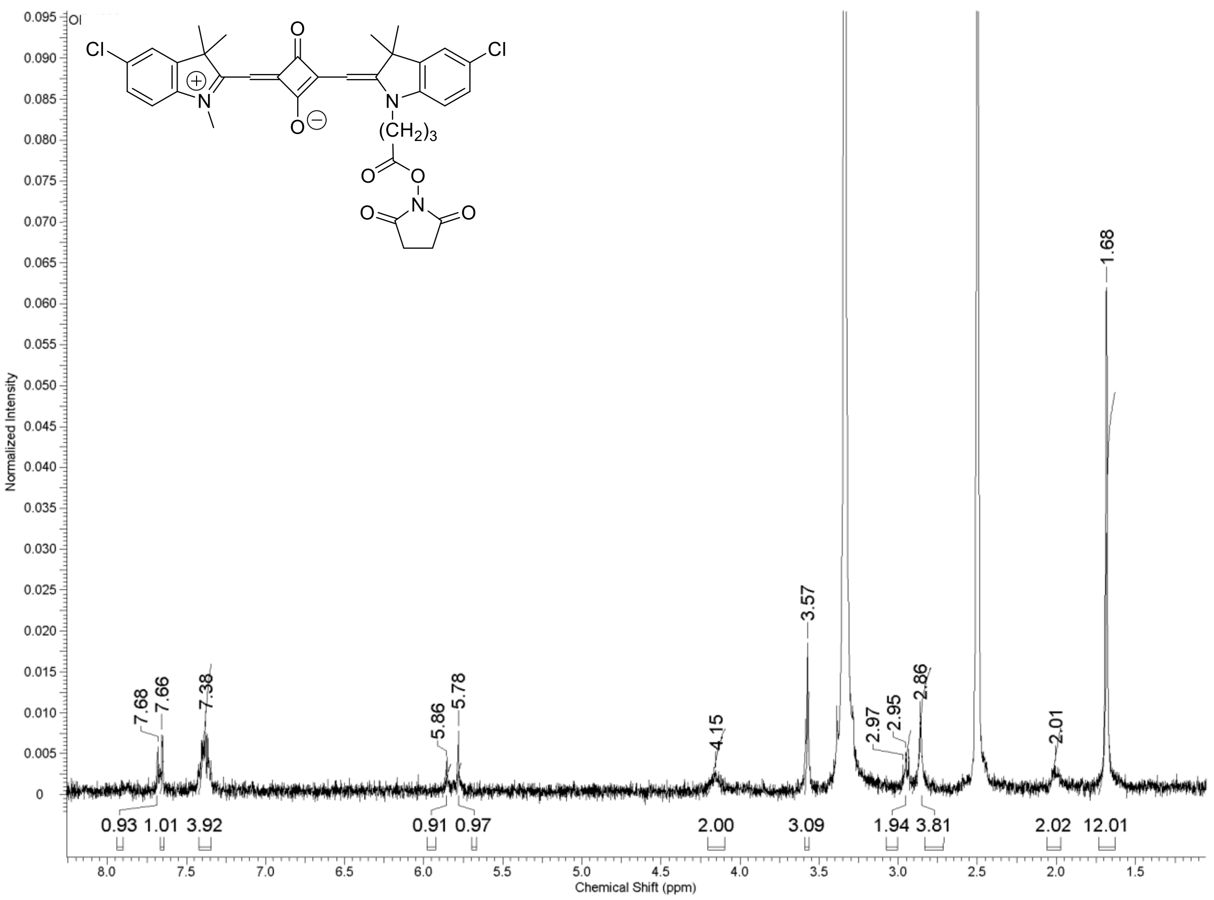

Figure S2. ^{1}H NMR spectrum of **SQ-C3-NHS** (400 MHz, DMSO-d_6).

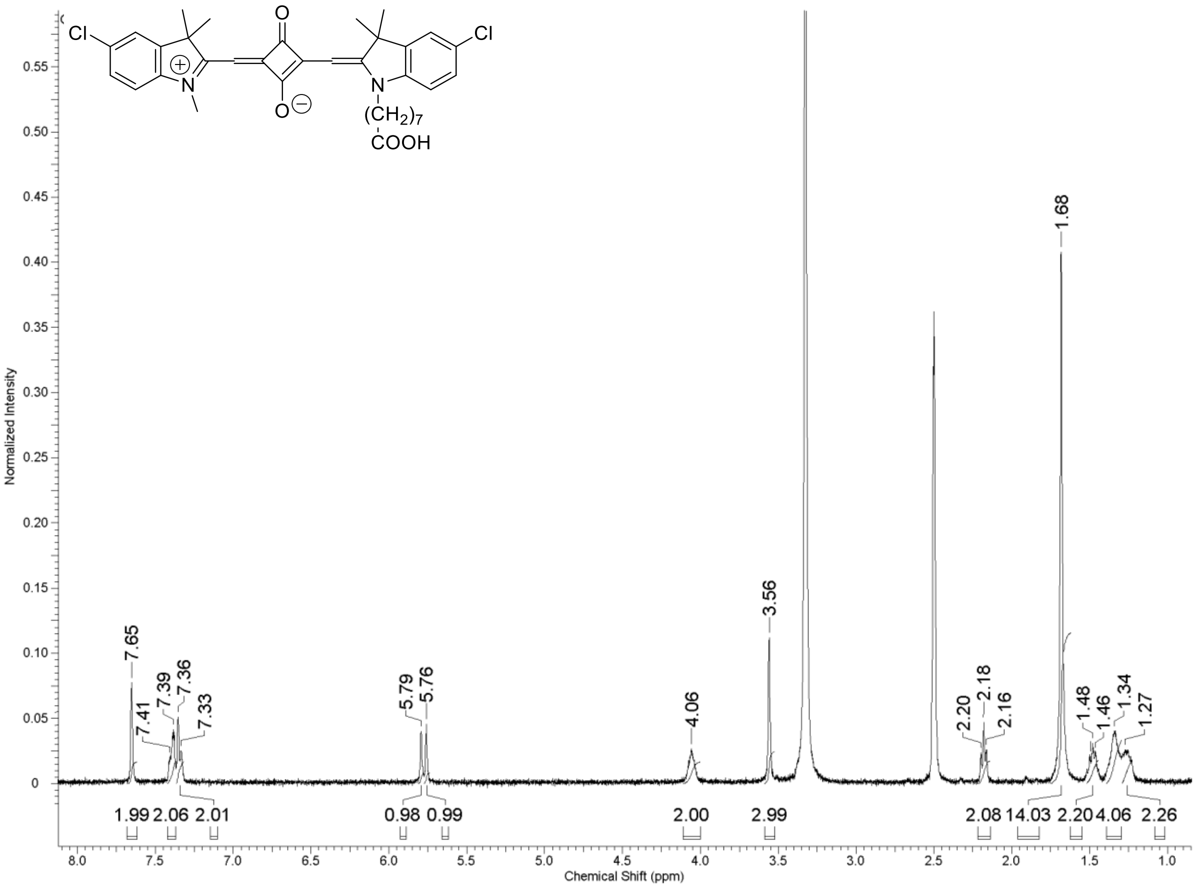

Figure S3. ^{1}H NMR spectrum of **SQ-C7-COOH** (400 MHz, DMSO-d_6).

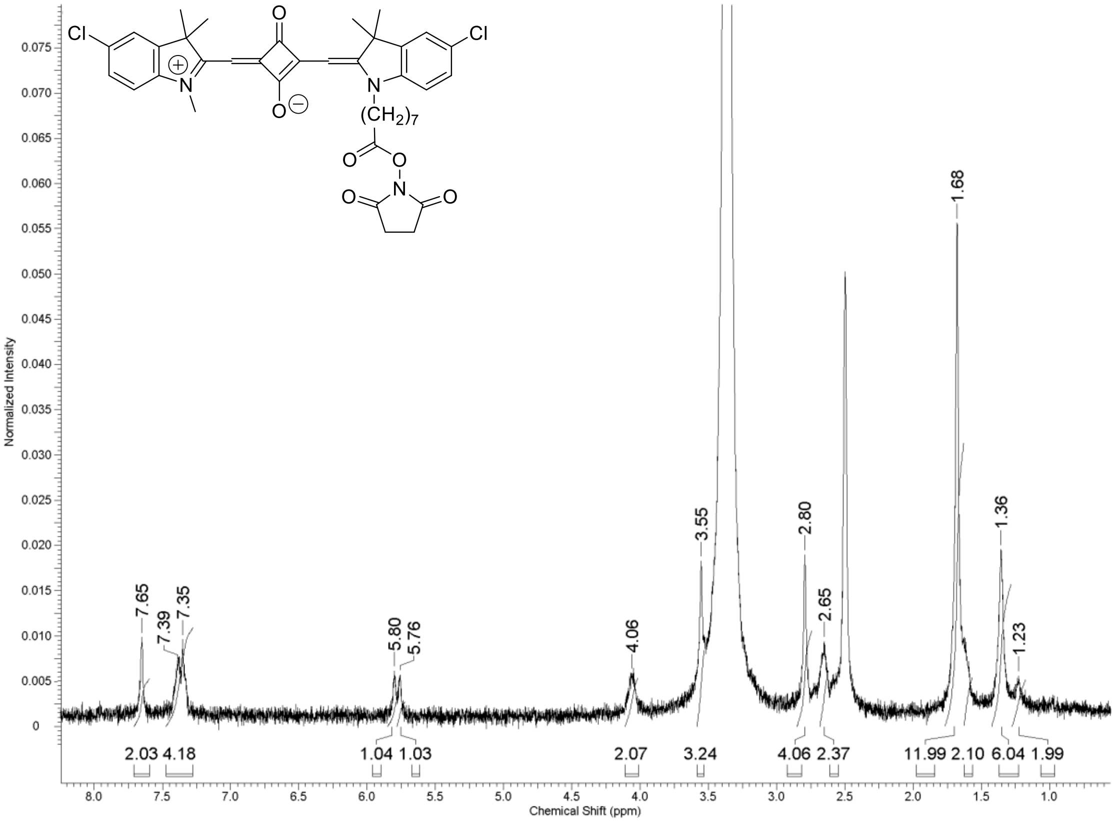

Figure S4. ^{1}H NMR spectrum of **SQ-C7-NHS** (400 MHz, DMSO-*d*$_6$).

Supporting Information 2: *Oligo Sequences*

Table S1. Oligo sequences used to assemble squaraine monomers and dimers templated by immobile HJ.

Strand Name	[a]Sequence (5′ to 3′)	Length (nt)	Purification
**A**	ATA TAA TCG CTC GC ATA TTA TGA CTG	26	Standard desalting
**B**	CAG TCA TAA TAT GT GGA ATG TGA GTG	26	Standard desalting
**C**	CAC TCA CAT TCC AC TCA ACA CCA CAA	26	Standard desalting
**D**	TTG TGG TGT TGA GC GAG CGA TTA TAT	26	Standard desalting
**SQ-R$_1$-A**	ATA TAA TCG CTC G[SQ-R$_1$-T]C ATA TTA TGA CTG	27	Dual HPLC
**SQ-R$_2$-A**	ATA TAA TCG CTC G[SQ-R$_2$-T]C ATA TTA TGA CTG	27	Dual HPLC
**SQ-R$_3$-A**	ATA TAA TCG CTC G[SQ-R$_3$-T]C ATA TTA TGA CTG	27	Dual HPLC
**SQ-R$_1$-B**	CAG TCA TAA TAT G[SQ-R$_1$-T]T GGA ATG TGA GTG	27	Dual HPLC
**SQ-R$_2$-B**	CAG TCA TAA TAT G[SQ-R$_2$-T]T GGA ATG TGA GTG	27	Dual HPLC
**SQ-R$_3$-B**	CAG TCA TAA TAT G[SQ-R$_3$-T]T GGA ATG TGA GTG	27	Dual HPLC
**SQ-R$_1$-C**	CAC TCA CAT TCC A[SQ-R$_1$-T]C TCA ACA CCA CAA	27	Dual HPLC
**SQ-R$_2$-C**	CAC TCA CAT TCC A[SQ-R$_2$-T]C TCA ACA CCA CAA	27	Dual HPLC
**SQ-R$_3$-C**	CAC TCA CAT TCC A[SQ-R$_3$-T]C TCA ACA CCA CAA	27	Dual HPLC

[a]complementary regions are color-coded.

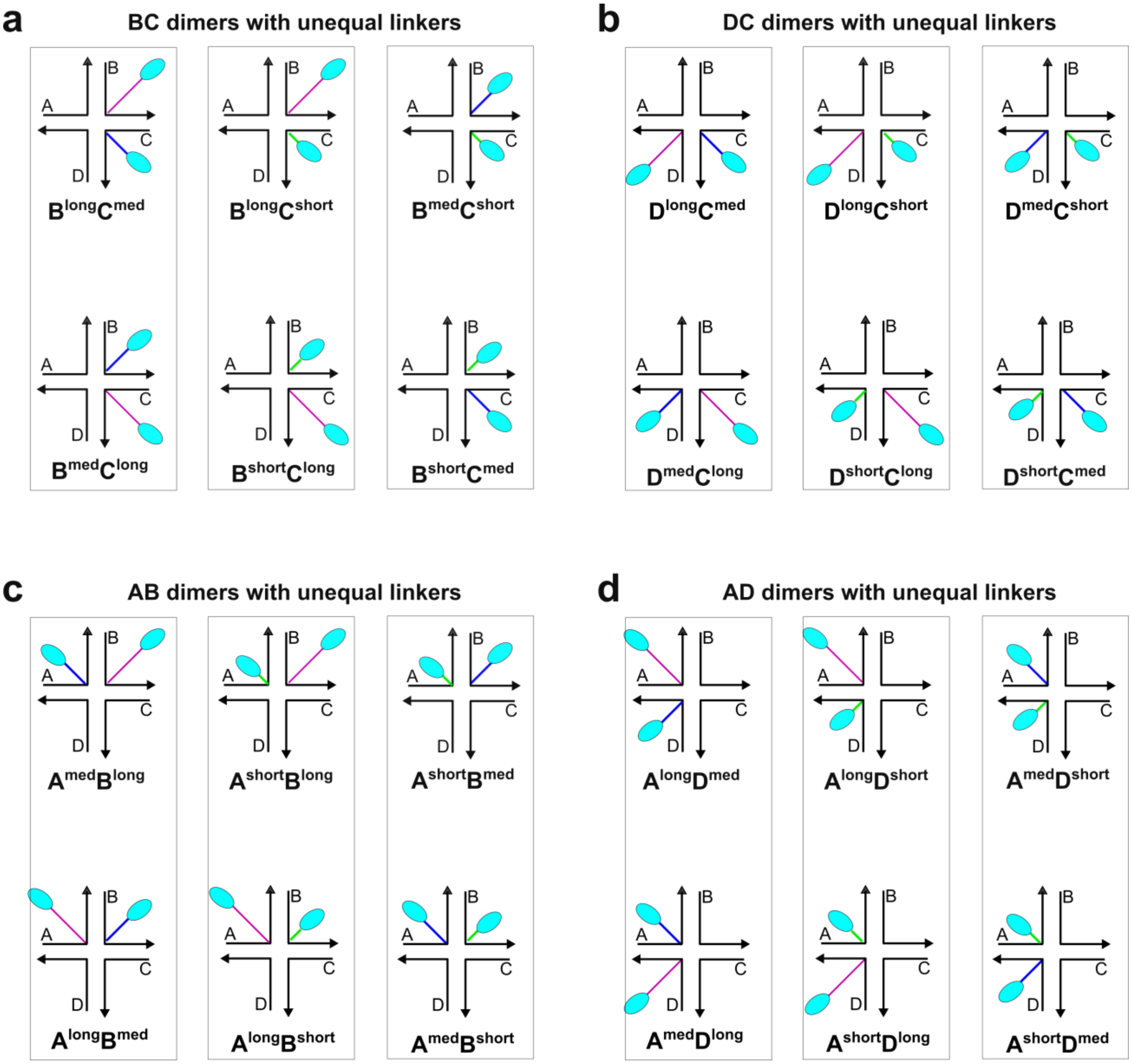

Figure S5. Schematics of squaraine dimers investigated for exciton chirality inversion.

Supporting Information 3: *Thermal Denaturation*

Melting profiles of squaraine dimers template by HJ were recorded in 1.0 cm quartz cuvettes at 260 nm using a Varian Cary5000 spectrophotometer equipped with a thermal probe. Samples were degassed for 5 min at room temperature (Centrivap DNA Vacuum Concentrator, Labconco). Samples were equilibrated at 22 °C for 5 min before starting a temperature ramp of 1 °C/min over a temperature range from 22 °C to 95 °C. Absorption was monitored at 260 nm. The thermal denaturation temperatures (T_m) were determined by fitting Gaussian into the first derivative of absorbance in OriginPro 2019.

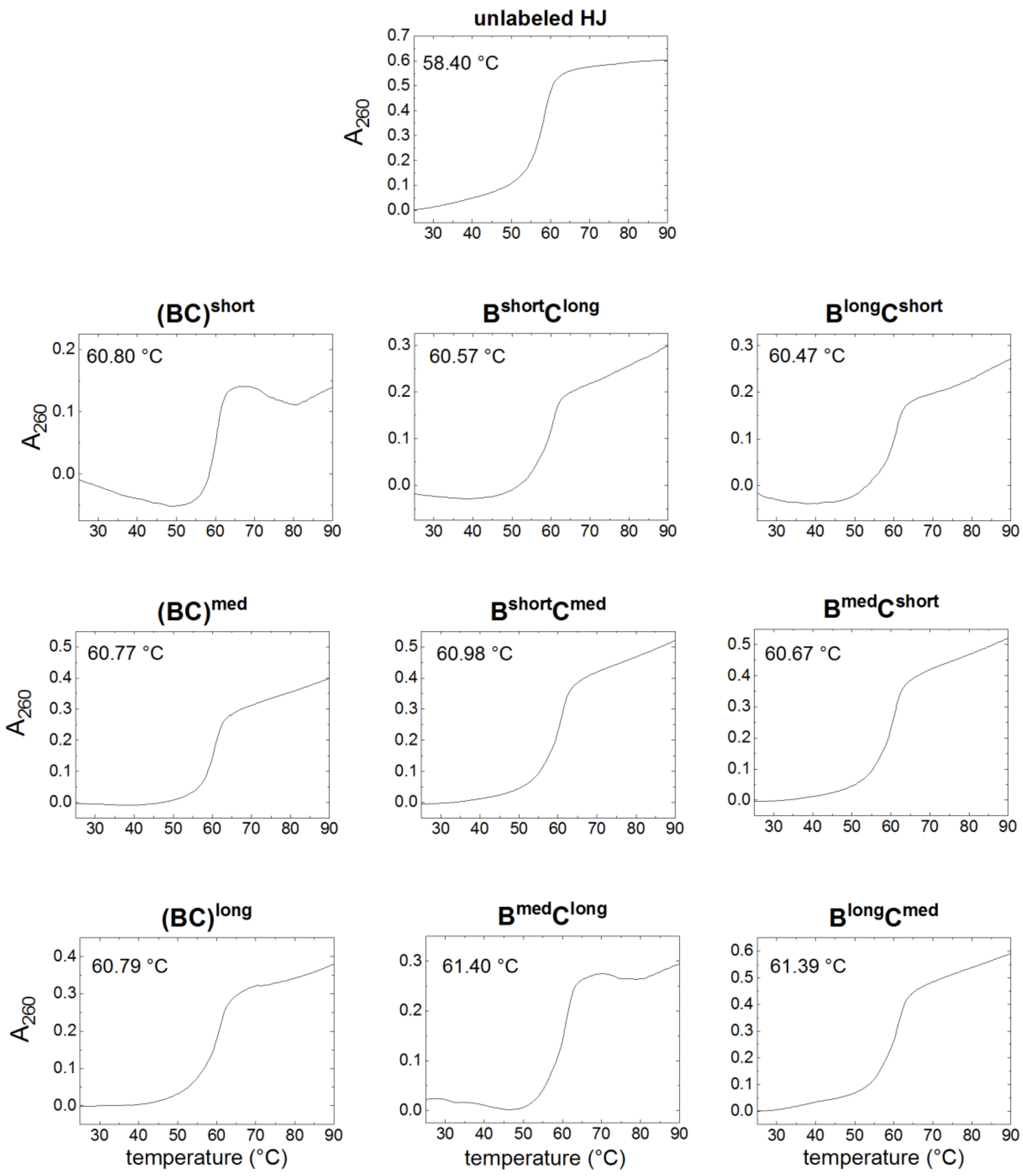

Figure S6. UV melting curves of unlabeled HJ (a control) and squaraine BC dimers at 260 nm in 1× TBE, 15 mM $MgCl_2$. The insets show melting temperatures determined from the maximum of the absorbance first derivative.

Supporting Information 4: *Electrophoretic Analysis*

Non-denaturing electrophoresis was performed to analyze the formation of squaraine dimers with linkers of unequal length. A non-denaturing 15% PAGE gel, 1.5 mm-thick, was cast in 1× TBE, 15 mM $MgCl_2$ buffer. DNA constructs were combined with a loading buffer [20% v/v Ficoll (Sigma-Aldrich) and 20% v/v bromophenol blue (Sigma-Aldrich)] to the final concentration 0.075 µM for single-stranded DNA, and 0.15 µM for unlabeled HJ and squaraine dimers. The electrophoresis was carried out at 17 °C, 150 V applied voltage in 1× TBE, 15mM $MgCl_2$ running buffer for 1 h 45 min. The gels were imaged in FluorChem Q imager (Alpha Innotech, San Leandro, CA) using the Cy5 channel (λ_{ex} = 632 nm, λ_{em} = 691 nm). Next, the gel was washed (3× 200 mL aqueous solution of 5% v/v EtOH, 5% v/v MeOH) and stained with SYBR Gold (Invitrogen). The stained gel was imaged using the Cy2-channel (λ_{ex} = 475 nm, λ_{em} = 506 nm). The resulting raw fluorescence gel images (i. e., without brightness/contrast adjustments) are shown in Figure S7.

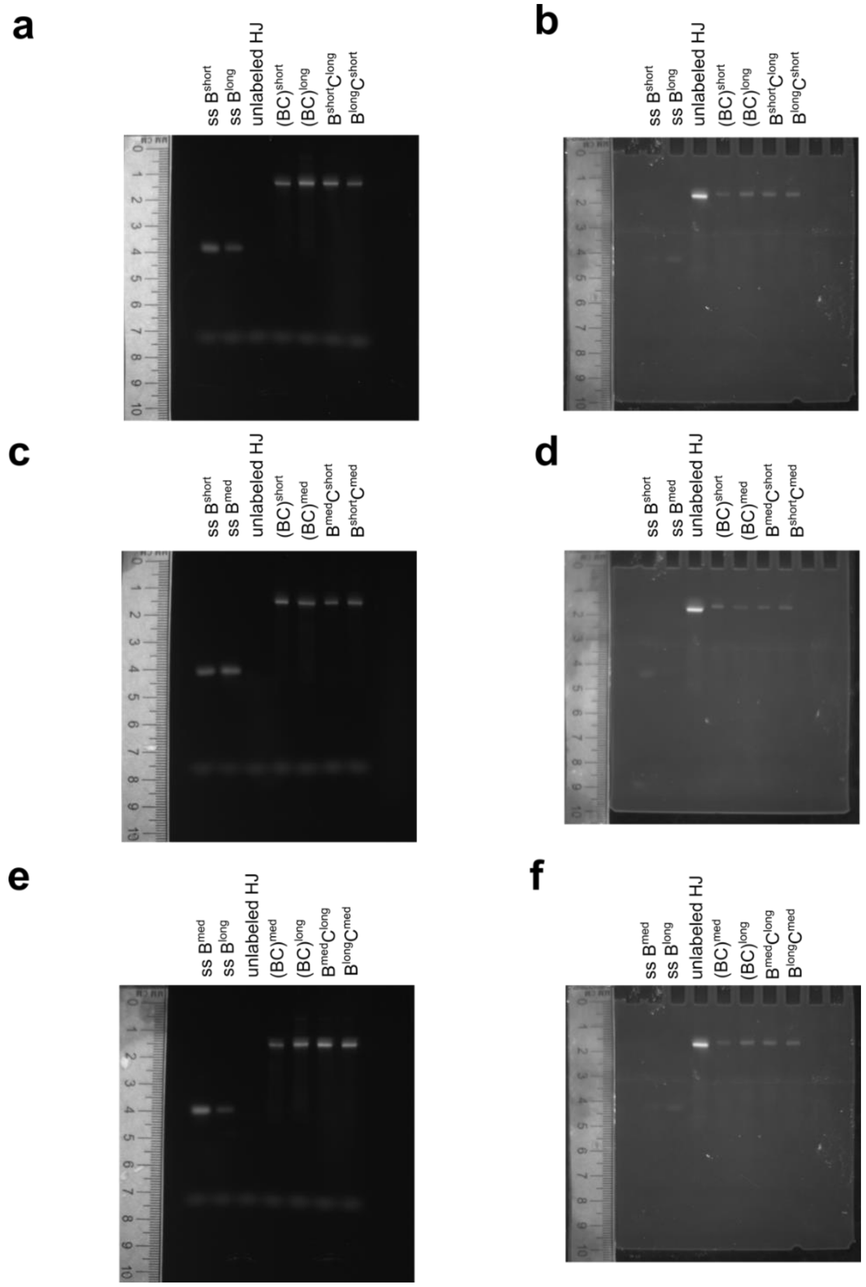

Figure S7. Fluorescence images of 15% non-denaturing PAGE, 1.5 mm-thick, of squaraine BC dimers with linkers of unequal length. The electrophoresis was performed at 17 °C, 150 V constant voltage in 1× TBE, 15 mM $MgCl_2$ running buffer. Single-stranded DNA labeled with squaraine dye, unlabeled HJ, and dimers with equal linkers were used as controls. Dimers and unlabeled HJ were applied on the gel at 0.15 μM concentration, and single strands were applied on the gel at 0.075 μM concentration. (**a**, **c**, **e**) Fluorescence image of non-stained gel under Cy5-channel (λ_{ex} = 632 nm, λ_{em} = 691 nm). Unlabeled HJ is not visible. The fluorescence of squaraine aggregates is suppressed due to aggregation. (**b**, **d**, **f**) Fluorescence image of SYBR Gold-stained gel under Cy2-channel (λ_{ex} = 475 nm, λ_{em} = 506 nm). Fluorescence of squaraine-labeled DNA is suppressed due to interference with SYBR Gold.

Supporting Information 5: *Full Absorption and CD Spectra of Squaraine Monomers and Dimers*

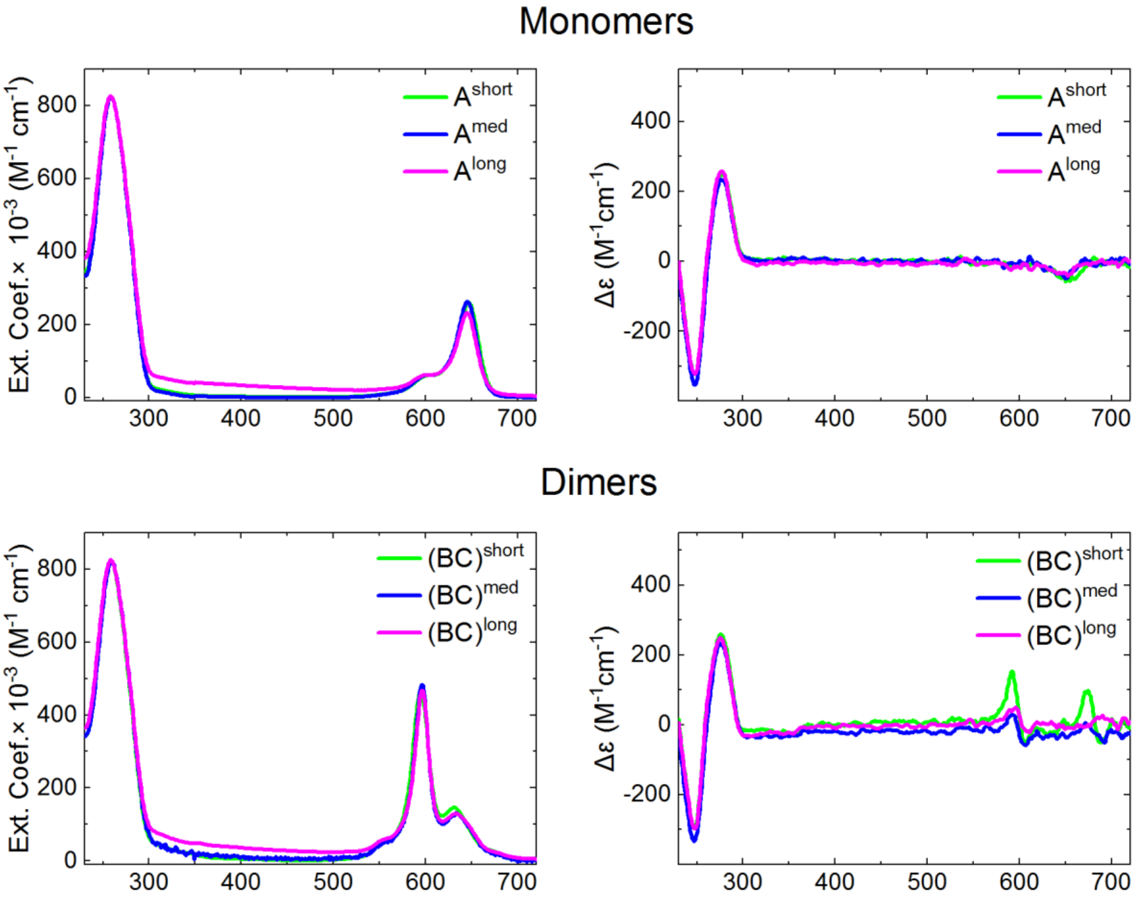

Figure S8. Steady-state absorption (left column) and CD (right column) spectra of squaraine monomers and dimers with equal linker length covalently templated by DNA HJ. The spectra were acquired at room temperature in 1× TBE, 15 mM $MgCl_2$ with the squaraine-DNA construct concentration 1.5 μM.

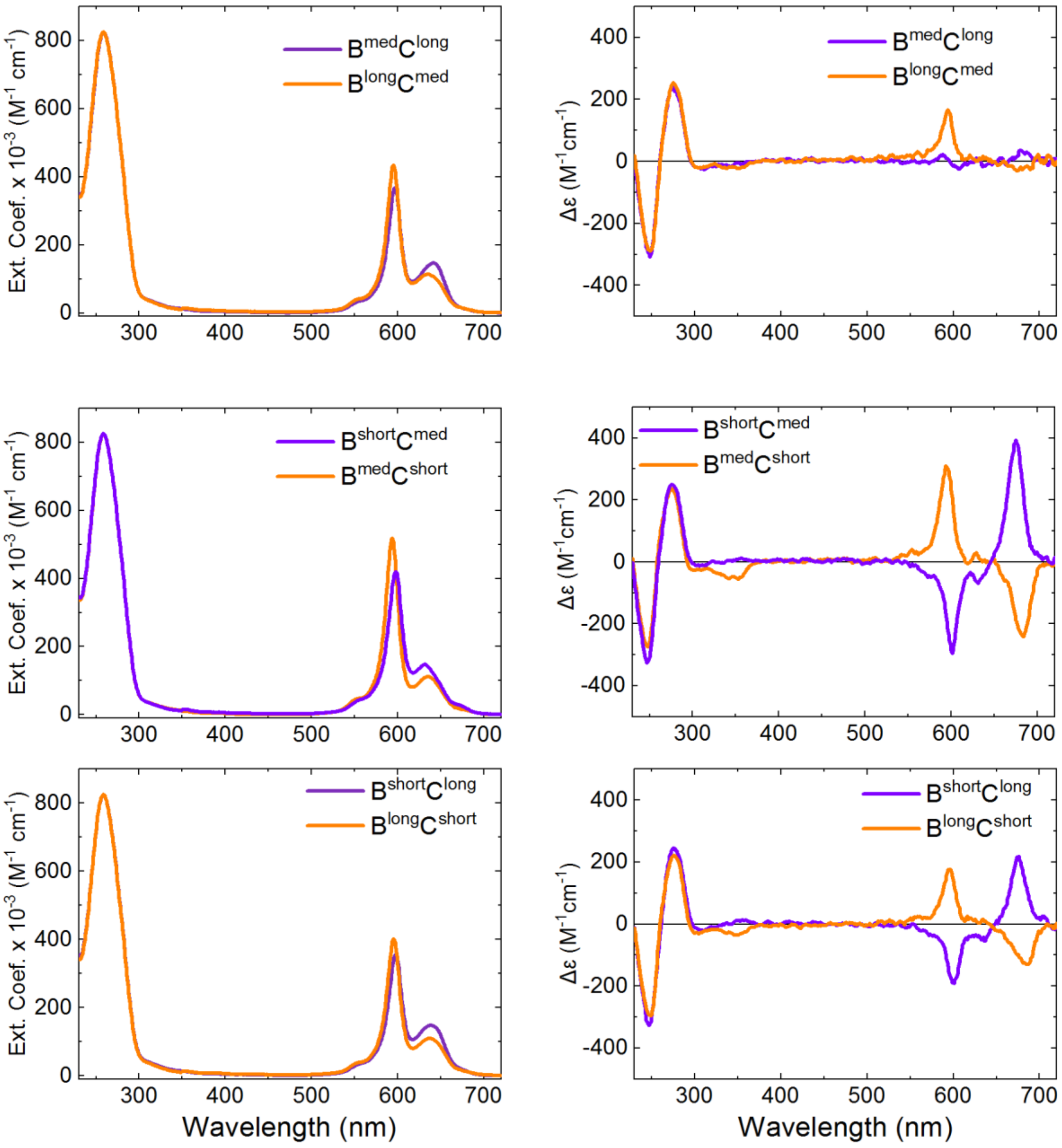

Figure S9. Steady-state absorption (left column) and CD (right column) spectra of squaraine monomers and dimers with unequal linker length covalently templated by DNA HJ. The spectra were acquired at room temperature in 1× TBE, 15 mM $MgCl_2$ with the squaraine-DNA construct concentration 1.5 μM.

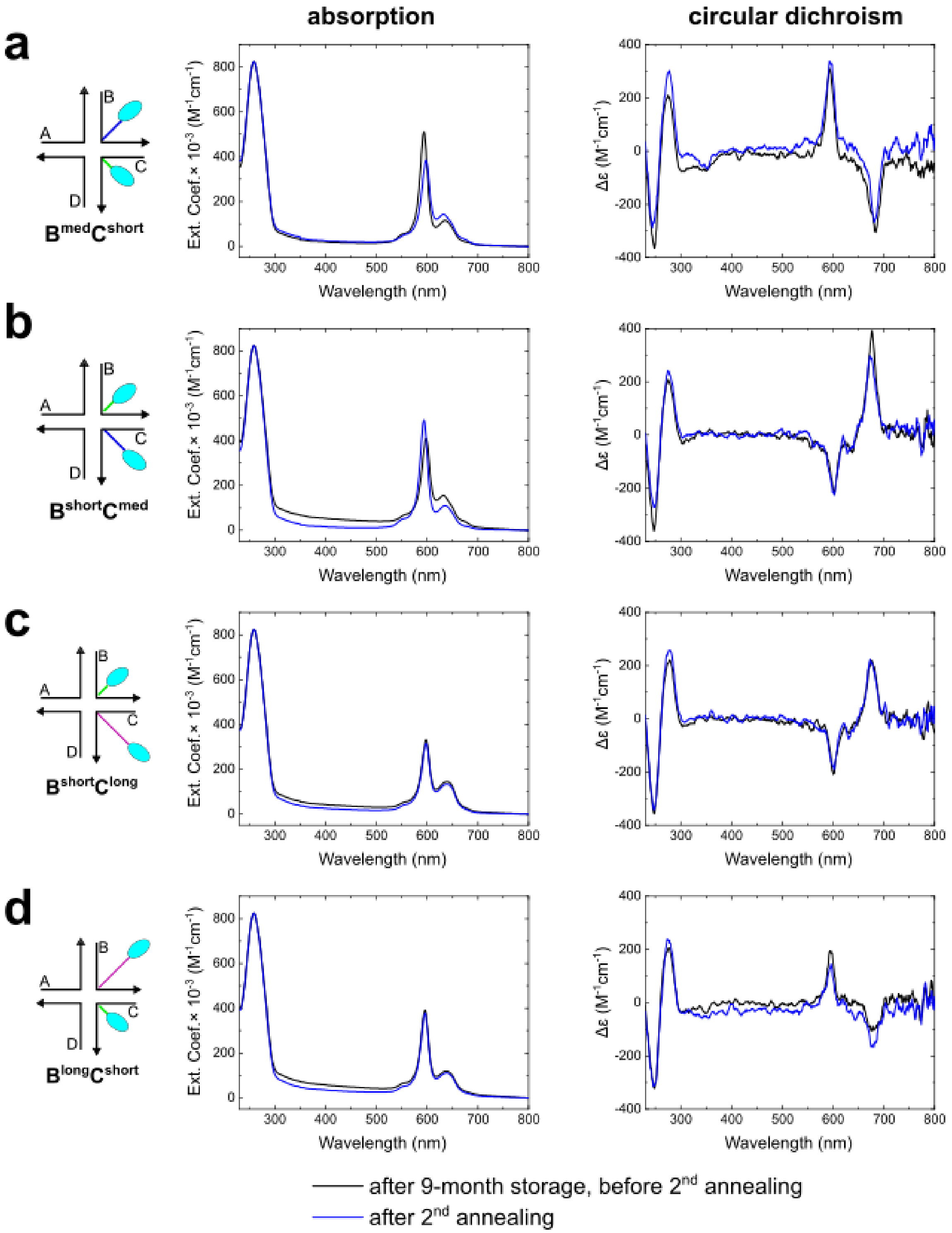

Figure S10. Steady-state absorption and CD spectra of re-annealed dimers with unequal linkers. After storage for nine months at 4 °C, dimers were re-annealed at 95 °C for 4 min, followed by cooling at 1 °C/min to room temperature.

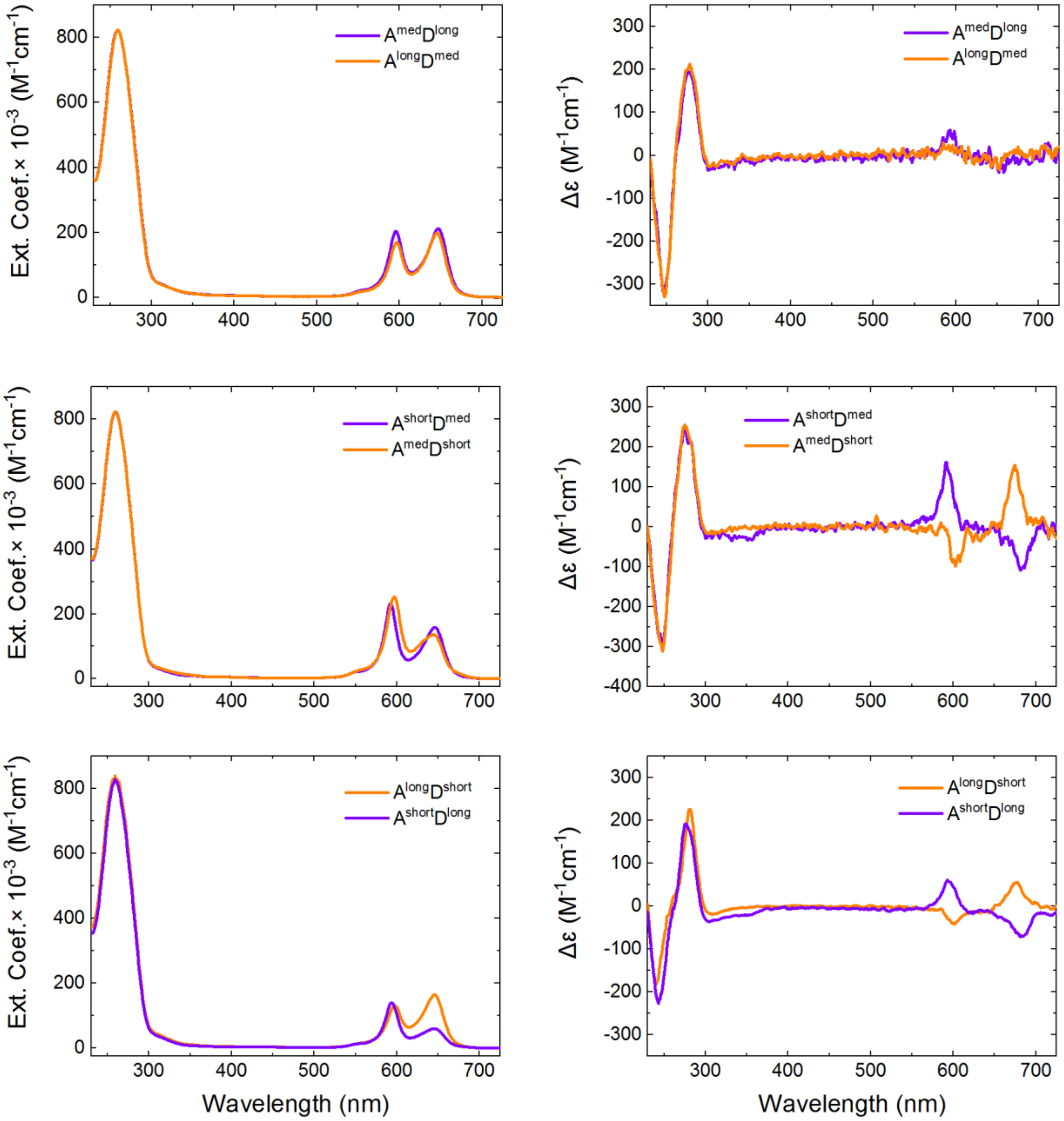

Figure S11. Steady-state absorbance (left column) and CD (right column) spectra of squaraine AD dimers with unequal linker length covalently templated by HJ. The spectra were acquired at room temperature in 1× TBE, 15 mM $MgCl_2$ with the squaraine-DNA construct concentration 1.5 - 4.0 μM. The samples of squaraine-labeled single strands used to prepare AD dimers contained a larger amount of unlabeled strands resulting in a larger monomer population in the dimer samples. The absorption spectra were normalized to the molar extinction coefficient. The CD spectra were normalized to molar CD.

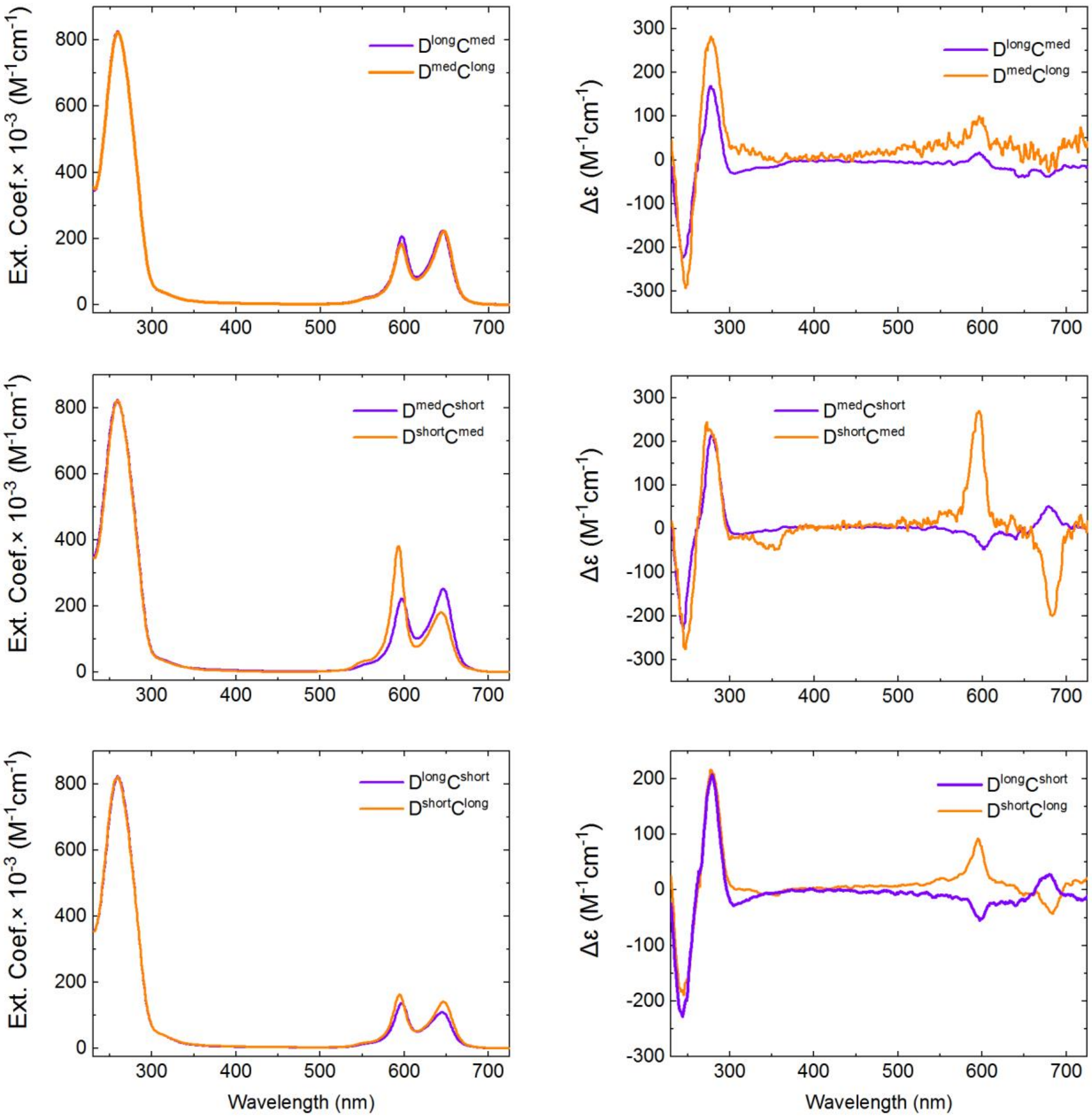

Figure S12. Steady-state absorbance (left column) and CD (right column) spectra of squaraine DC dimers with unequal linker length covalently templated by HJ. The spectra were acquired at room temperature in 1× TBE, 15 mM $MgCl_2$ with the squaraine-DNA construct concentration 1.5 - 4.0 μM. The samples of squaraine-labeled single strands used to prepare DC dimers contained a larger amount of unlabeled strands resulting in a larger monomer population in the dimer samples. The absorption spectra were normalized to the molar extinction coefficient. The CD spectra were normalized to molar CD.

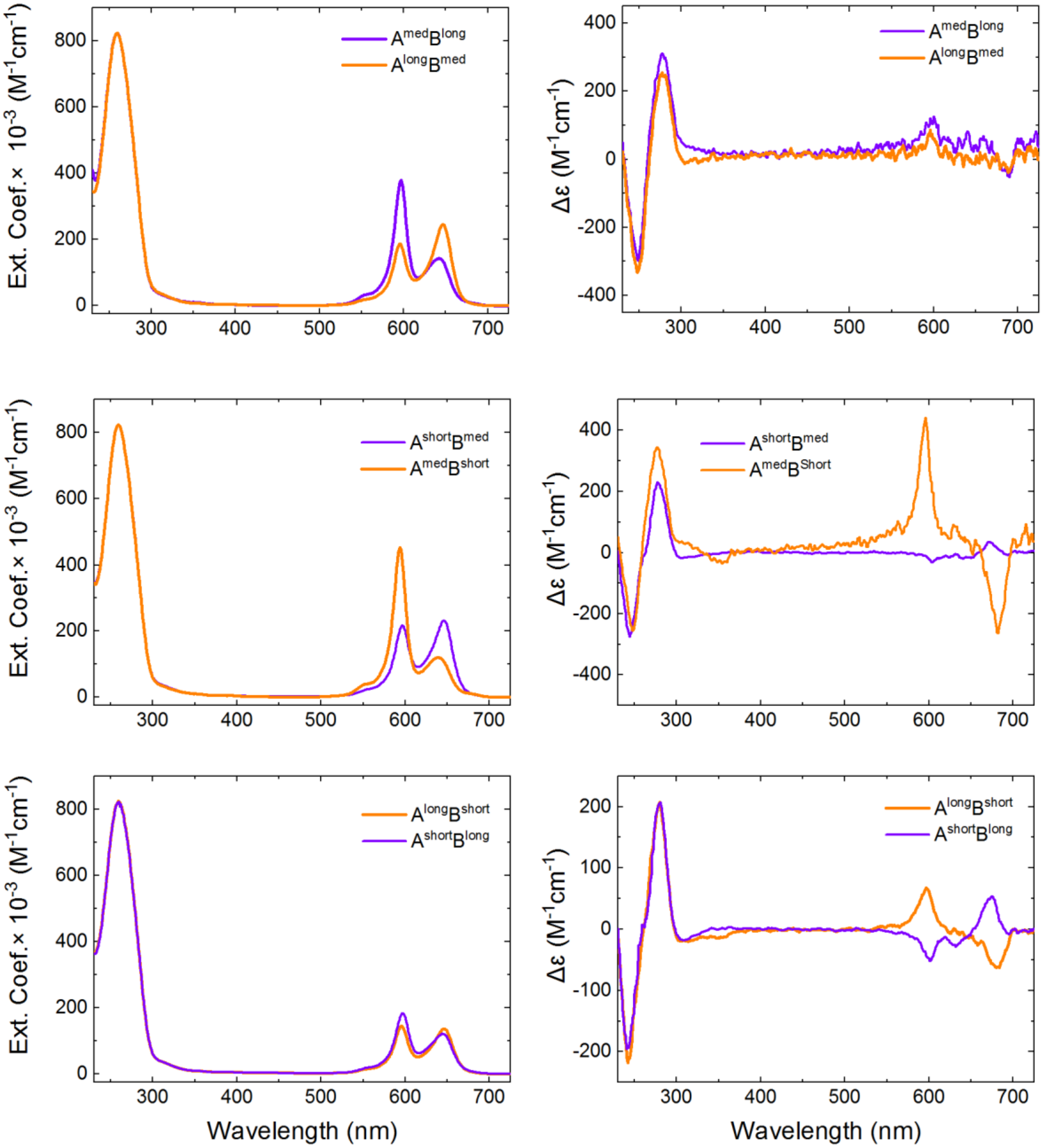

Figure S13. Steady-state absorbance (left column) and CD (right column) spectra of squaraine AB dimers with unequal linker length covalently templated by DNA HJ. The spectra were acquired at room temperature in 1× TBE, 15 mM $MgCl_2$ with the squaraine-DNA construct concentration 1.5 - 4.0 μM. The samples of squaraine-labeled single strands used to prepare AB dimers contained a larger amount of unlabeled strands resulting in a larger monomer population in the dimer samples. The absorption spectra were normalized to the molar extinction coefficient. The CD spectra were normalized to molar CD.

Supporting Information 6: *Fitting Absorption and CD using KRM Model*

When intermolecular distances are much greater than dye size, the excitonic hopping parameter $J_{m,n}$ can be expressed in a point dipole-dipole interaction form[4-6] representing the interaction between a pair of molecular transition dipoles μ_m and μ_n for the dyes at sites *m* and *n*:

$$J_{m,n} = \frac{1}{4\pi\varepsilon\varepsilon_0}\left(\frac{\boldsymbol{\mu_m}\cdot\boldsymbol{\mu_n}}{|\boldsymbol{R_{m,n}}|^3} - \frac{(\boldsymbol{\mu_m}\cdot\boldsymbol{R_{m,n}})(\boldsymbol{\mu_n}\cdot\boldsymbol{R_{m,n}})}{|\boldsymbol{R_{m,n}}|^5}\right) \quad \text{(eq S1)}$$

where $\boldsymbol{R_{m,n}}$ – the position vector between the dye centers dyes *m* and *n*. (further $\boldsymbol{R_{1,2}}$ between two dyes)

In the extended dipole approximation,[7] the Coulomb energy between a pair of dipoles is given in Standard International units by:

$$J_{m,n} = \frac{\delta^2}{4\pi\epsilon_0\epsilon_r}\left(\frac{1}{|\boldsymbol{r_1}-\boldsymbol{r_2}|} - \frac{1}{|\boldsymbol{r_1}-\boldsymbol{s_2}|} - \frac{1}{|\boldsymbol{s_1}-\boldsymbol{r_2}|} + \frac{1}{|\boldsymbol{s_1}-\boldsymbol{s_2}|}\right) \quad \text{(eq S2)}$$

where δ is the oscillating point charge in Coulombs, ϵ_0 is the permittivity of the vacuum (ϵ_0 = 8.85 × $10^{-12}m^{-3}kg^{-1}s^4A^2$), ϵ_r is the relative dielectric constant of the medium, $\boldsymbol{r_1}$ and $\boldsymbol{s_1}$ are the location of the "+" and "- "charges on molecule *1* and similarly for molecule *2*. The distance units are meters and the exchange energy $J_{m,n}$ unit are Joules.

The transition dipole moment is given by $\mu = \delta l$ where l is the distance in meters between the two point charges on a given molecule, and *r* is given by $\epsilon_r = n^2$ where *n* is the index of refraction of the medium.

Then the equation (S2) can be rewritten as:

$$J_{12} = \frac{J_0}{l^2}\left(\frac{1}{|\boldsymbol{r_1}-\boldsymbol{r_2}|} - \frac{1}{|\boldsymbol{r_1}-\boldsymbol{s_2}|} - \frac{1}{|\boldsymbol{s_1}-\boldsymbol{r_2}|} + \frac{1}{|\boldsymbol{s_1}-\boldsymbol{s_2}|}\right) \quad \text{(eq S3)}$$

where $J_0 = \frac{\mu^2}{4\pi\epsilon_0 n^2}$ (eq S4)

The quantity J_0 has units of $J{\cdot}m^3$.

When the position vectors $\boldsymbol{r}$ and $\boldsymbol{s}$ are rewritten as:

$$\boldsymbol{r_1} = \boldsymbol{R_1} + \frac{l}{2}\boldsymbol{n_1} \qquad \boldsymbol{s_1} = \boldsymbol{R_1} - \frac{l}{2}\boldsymbol{n_1} \qquad \boldsymbol{r_2} = \boldsymbol{R_2} + \frac{l}{2}\boldsymbol{n_2} \qquad \boldsymbol{s_2} = \boldsymbol{R_2} - \frac{l}{2}\boldsymbol{n_2}$$

And when $|\boldsymbol{R_1} - \boldsymbol{R_2}| \gg l$, equation (eq S3) reduces to:

$$J_{12} = \frac{J_0}{|\boldsymbol{R_1}-\boldsymbol{R_2}|^3}[\boldsymbol{n_1}\cdot\boldsymbol{n_2} - 3(\boldsymbol{n_{12}}\cdot\boldsymbol{n_1})(\boldsymbol{n_{12}}\cdot\boldsymbol{n_2})] \quad \text{(eq S5)}$$

where $\boldsymbol{n_{12}} = \frac{\boldsymbol{R_1}-\boldsymbol{R_2}}{|\boldsymbol{R_1}-\boldsymbol{R_2}|}$

Thus, J_0 is the same constant in both the point dipole-dipole approximation and in the extended dipole approximation.

The theoretical absorbance as a function of energy, for comparison with experimental data, was computed from the line spectra obtained by diagonalizing the system Holstein-like Hamiltonian[8] by convolution with a Gaussian as:

$$A(E) = \sum_i \frac{\gamma_i}{\sqrt{2\pi\Gamma^2}} exp\ \left(-\frac{(E-E_i)^2}{2\Gamma^2}\right) \quad \text{(eq S6)}$$

Where the γ_i is the transition rates between the ground state E and the state having the eigenenergy E_i, and Γ is a linewidth.

Similarly, the CD absorbance as a function of energy was computed as:

$$A_{CD}(E) = \sum_i \frac{\gamma_i^{CD}}{\sqrt{2\pi\Gamma^2}} exp\left(-\frac{(E-E_i)^2}{2\Gamma^2}\right) \quad \text{(eq S7)}$$

The phenomenological constants used in the theoretical model are shown in **Table S2.** To fit the monomer and then dimers and a tetramer data, we considered the length of the squaraine transition dipole moment to be 1.3 nm (which is slightly shorter than the length of an indolenine squaraine dye), the linewidth Γ to be 0.028-0.034 eV, and number of vibronic states n_v to be 3.

The transition dipole moment was determined using the following expression:[9]

$$M_{01} = 9.58 \times 10^{-2}\left(\frac{\left(2n^2+1\right)^2}{9n^3}\int \frac{\epsilon(\nu)}{\nu}d\nu\right)^{\frac{1}{2}} \quad \text{(eq S8)}$$

M_{01} – transition dipole moment in debye; n - refractive index of water (n_{water} = 1.3327).

Note that μ has units of Coulomb meters while M_{01} has units of debye. The conversion relation between the two is 1 debye = 3.33564 × 10^{-30} C·m.

The values of characteristic exciton hopping parameter J_0 calculated from M_{01} according to the equation (S4) are reported in **Table S3**, and its values were used as a fitting parameter in the calculations of squaraine dimers.

As a measure of the goodness of fit, we evaluated the overlap integrals of the experimental spectra with the theoretical spectra (**Table S4**). Letting $\int S_{ab,ex}(E), S_{ab,th}(E)$, $S_{cd,ex}(E), and\ S_{cd,th}(E)$ denote respectively the experimental absorbance spectrum, theoretical absorbance spectrum, experimental CD spectrum, and theoretical CD spectrum where ***E*** is energy, the normalized absorbance overlap integral OI_{AB} of the spectrum is defined by:

$$OI_{AB} = \frac{\int S_{ab,ex}(E)S_{ab,th}(E)dE}{\sqrt{\int S_{ab,ex}^2(E)dE}\sqrt{\int S_{ab,th}^2(E)dE}} \quad \text{(eq S9)}$$

and the normalized overlap integral for the CD spectrum OI_{CD} is defined by:

$$OI_{CD} = \frac{\int S_{cd,ex}(E)S_{cd,th}(E)dE}{\sqrt{\int S_{cd,ex}^2(E)dE}\sqrt{\int S_{cd,th}^2(E)dE}} \quad \text{(eq S10)}$$

As an overall goodness parameter, we introduce:

$$OI_{tot} = \frac{1}{2}(OI_{ab} + OI_{cd}) \quad \text{(eq S11)}$$

In addition, the mean-square deviation of absorbance and CD were utilized:

$$ms_{abs} = \sum_i [S_{ab,ex}(E) - S_{ab,th}(E)]^2 \qquad \text{(eq S12)}$$

$$ms_{cd} = \sum_i [S_{cd,ex}(E) - S_{cd,th}(E)]^2 \qquad \text{(eq S13)}$$

$$Fitness = w_1(1-r)^2 + w_2(1-OI_{AB})^2 + w_3(1-OI_{CD})^2 + w_4 ms_{abs} + w_5 ms_{cd} \qquad \text{(eq S14)}$$

where w_1, w_2, w_3, and w_4 are user selected weights. For a typical run the weights were chosen as $w_1 = 1$, $w_2 = w_3 = 0$, and $w_4 = w_5 = 1$.

The resulting outputs of the fit provide information regarding the angles and position of the dyes relative to each other. Given in spherical coordinates, the zenith (θ_i) and azimuth (φ_i) angles are given in degrees. The Cartesian components of the orientation vector for a dye are given in terms of θ_i and φ_i by the following set of equations:

$$n_x = sin\,(\theta_i)\,cos\,(\varphi_i)$$

$$n_y = sin\,(\theta_i)\,sin\,(\varphi_i)\,,$$

$$n_z = cos\,(\theta_i).$$

The positions of the dyes are given in nm and listed in **Table S5.**

A center-to-center distance R between two dyes was calculated as:

$$R = \sqrt{(x_2 - x_1)^2 + (y_2 - y_1)^2 + (z_2 - z_1)^2}.$$

A slip angle θ_s was calculated as:

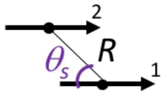

$$\theta_s = cos^{-1}[\boldsymbol{n_1} \cdot \boldsymbol{n_{12}}]$$

Where $\boldsymbol{n_1}$ is a unit orientation vector given by:

$$\boldsymbol{n_1} = sin(\theta_1)\cos(\varphi_1)\hat{x} + sin(\theta_1)\sin(\varphi_1)\hat{y} + \cos(\theta_1)\hat{z}$$

And where $\boldsymbol{n_{12}}$ is a unit vector connecting dye centers is:

$$\boldsymbol{n_{12}} = \frac{1}{R_{1,2}}[(x_2 - x_1)\hat{x} + (y_2 - y_1)\hat{y} + (z_2 - z_1)\hat{z}]$$

The oblique angle between two vectors was calculated as:

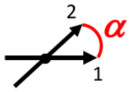

$$\alpha = cos^{-1}[\,\sin(\theta_1)\sin(\theta_2)\cos(\varphi_1)\cos(\varphi_2) + \sin(\theta_1)\sin(\theta_2)\sin(\varphi_1)\sin(\varphi_2) + \cos(\theta_1)\cos(\theta_2)].$$

Extracted $J_{1,2}$ values and calculated geometric parameters for dye aggregates are summarized in the **Table S6**.

Table S2. Input fitting parameters used in calculations of squaraine dimers with unequal linker length.

Parameter	Monomer		
	A^{short}	A^{med}	A^{long}
The energy of vibron E_v (*eV*)	0.150	0.155	0.170
Displacement of excited state vibronic potential *d* (dimensionless units)	0.66	0.66	0.64
Energy loss damping constant Γ (*eV*)	0.03	0.0315	0.028

Parameter	Dimer BC with Unequal Linker Length					
	$B^{short}C^{med}$	$B^{med}C^{short}$	$B^{short}C^{long}$	$B^{long}C^{short}$	$B^{med}C^{long}$	$B^{longCmed}$
Vibrational state Hilbert space n_v	3	3	3	3	3	3
Characteristic exciton hopping parameter J_0^* ($eV{\cdot}nm^3$)	0.05129	0.05129	0.05263	0.05263	0.05228	0.05228
Energy offset from monomer E_{of} (*eV*)	0.011	0.009	0.012	0.009	0.011	-0.001
Energy of vibron E_v (*eV*)	0.1525	0.1525	0.1625	0.1625	0.16	0.16
Displacement of excited state vibronic potential *d* (dimensionless units)	0.66	0.66	0.65	0.65	0.65	0.65
Energy loss damping constant Γ (*eV*)	0.03075	0.03075	0.02975	0.02975	0.029	0.029
Length of the transition dipole moment (*nm*)	1.4	1.4	1.4	1.4	1.4	1.4
Closest distance between the long axes of any pair of dyes (*nm*)	0.34	0.34	0.34	0.34	0.34	0.34

*J_0 was calculated from the fitting monomer absorption spectrum and used as an input parameter in the theoretical fitting of dimers and a tetramer.

Table S3. Output parameters extracted from calculations of the monomer templated in DNA HJ.

Parameter	A^{short}	A^{med}	A^{long}
Dipole moment M_{01} (*debye*)	12.1216	12.0402	12.3530
Characteristic exciton hopping parameter J_0^* ($eV \cdot nm^3$)	0.05164	0.0509	0.05363

Table S4. The goodness of the fit parameters for absorbance and CD spectra of squaraine dimers with unequal linker length.

Construct	*r*	OI_{AB}	OI_{CD}	OI_{Tot}	MSD_{abs}	MSD_{cd}	$w_{abscd}rms$
**$B^{short}C^{med}$**	1.00	0.98	0.96	0.97	0.18	0.50	0.68
**$B^{med}C^{short}$**	1.00	0.98	0.97	0.97	0.16	0.51	0.67
**$B^{short}C^{long}$**	1.00	0.97	0.96	0.96	0.33	0.49	0.82
**$B^{long}C^{short}$**	1.00	0.97	0.95	0.96	0.22	0.60	0.82
**$B^{med}C^{long}$**	1.00	0.97	0.44	0.70	0.33	5.91	0.33
**$B^{long}C^{med}$**	1.00	0.96	0.69	0.83	0.30	3.56	3.85

r - the ratio of theoretical to experimental values of the ratio of the max abs CD peak height to max absorbance peak height
OI_{AB} - normalized overlap integral for the experimental and theoretical absorbance curves
OI_{CD} - normalized overlap integral for the experimental and theoretical CD spectra
OI_{Tot} - mean of OI_{AB} and OI_{CD}
MSD_{abs} - absorbance spectrum mean-square deviation
MSD_{cd} - CD spectrum mean-square deviation
$w_{abscd}rms$ - weighted mean-squared deviation between the experimental and theoretical ABS and CD spectra

Table S5. Kühn-Renger-May model fitting outputs describing each dye orientation and position in dimer BC with for unequal linker lengths.

Dye	θ_i (°)	φ_i (°)	x_i (nm)	y_i (nm)	z_i (nm)
**$B^{short}C^{med}$**					
**Dye 1**	87.45	1.72	2.050	0.472	0.635
**Dye 2**	85.40	-9.83	2.047	0.084	0.418
**$B^{med}C^{short}$**					
**Dye 1**	95.81	-2.16	1.93	-0.272	1.056
**Dye 2**	89.11	-3.46	1.86	0.055	1.180
**$B^{short}C^{long}$**					
**Dye 1**	80.44	-0.72	1.228	-0.217	1.356
**Dye 2**	91.01	-1.05	1.310	-0.035	0.943
**$B^{long}C^{short}$**					
**Dye 1**	92.16	-3.32	2.239	-0.350	1.379
**Dye 2**	93.07	2.46	2.203	-0.633	1.121
**$B^{med}C^{long}$**					
**Dye 1**	88.63	-0.36	1.620	-0.225	1.303
**Dye 2**	97.09	3.15	1.561	-0.018	0.897
**$B^{long}C^{med}$**					
**Dye 1**	95.87	2.40	1.476	-0.271	0.891
**Dye 2**	93.81	6.60	1.379	-0.037	0.639

Table S6. Calculated $J_{1,2}$ and geometric parameters of dimer BC with unequal linker lengths.

Dimer Aggregate	$J_{1,2}$ (*meV*)	Center-to-Center distance *R*, (*Å*)	${}^{a}d_{min}$ (*nm*)	Slip angle (θ_s) °	Oblique angle (α) °
$\mathbf{B^{short}C^{med}}$	96.6	4.45	0.34	86.8<br>84.1	11.7
$\mathbf{B^{med}C^{short}}$	121.1	3.56	0.34	74.7<br>75.8	6.8
$\mathbf{B^{short}C^{long}}$	97.0	4.59	0.34	88.8<br>79.2	10.6
$\mathbf{B^{long}C^{short}}$	116.4	3.84	0.34	88.5<br>84.9	5.8
$\mathbf{B^{med}C^{long}}$	96.7	4.60	0.34	81.2<br>89.7	9.2
$\mathbf{B^{long}C^{med}}$	125.7	3.57	0.34	80.2<br>81.6	4.7

[a]the shortest distance between TDM vectors 1 and 2.

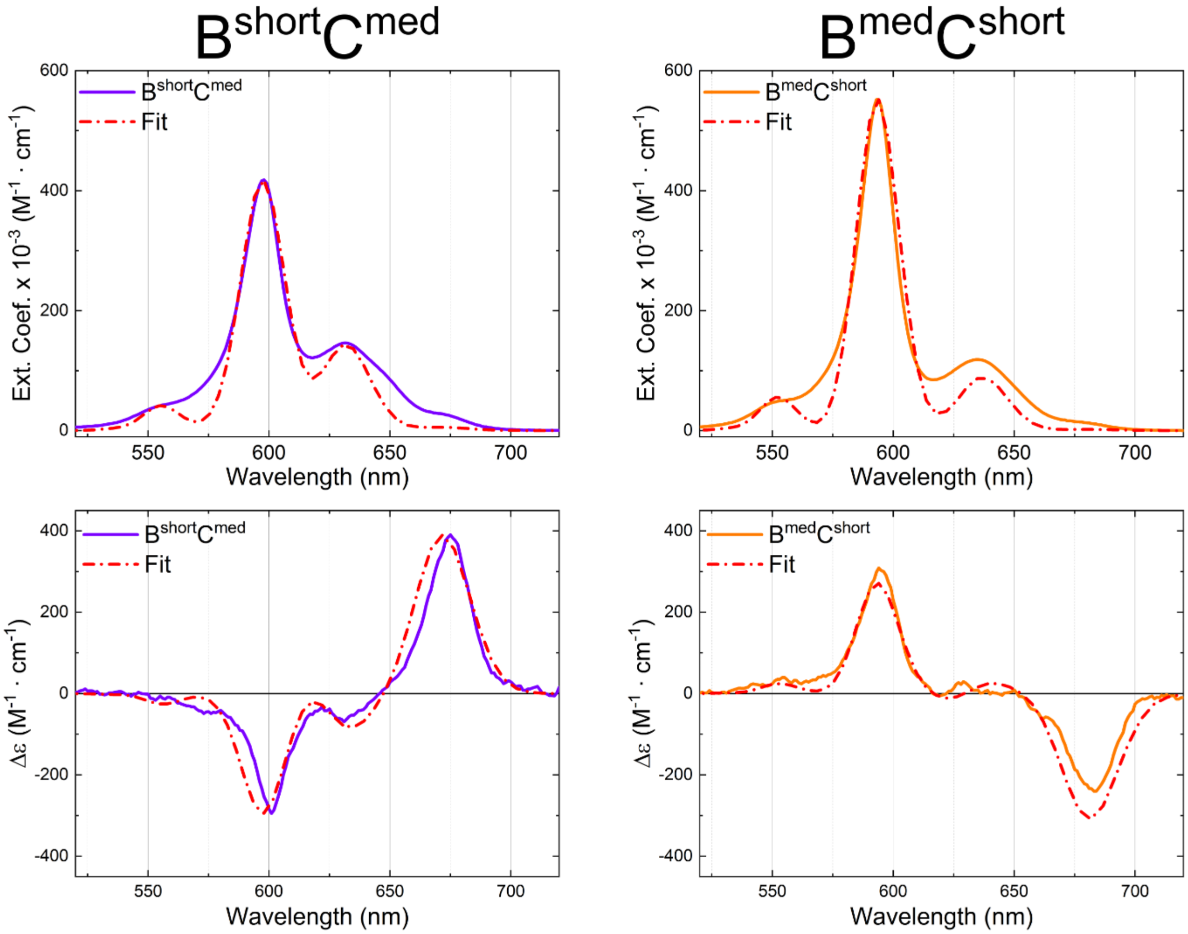

Figure S14. Acquired steady-state absorption and CD spectra (solid line) of squaraine dimers with unequal short and medium linker lengths covalently templated by DNA HJ with corresponding KRM Fit (dash-dot line). The spectra were recorded at room temperature with the squaraine-DNA construct concentration 1.5 μM in 1× TBE, 15 mM $MgCl_2$. The absorption spectra were converted to the molar extinction. The CD spectra were converted to molar CD.

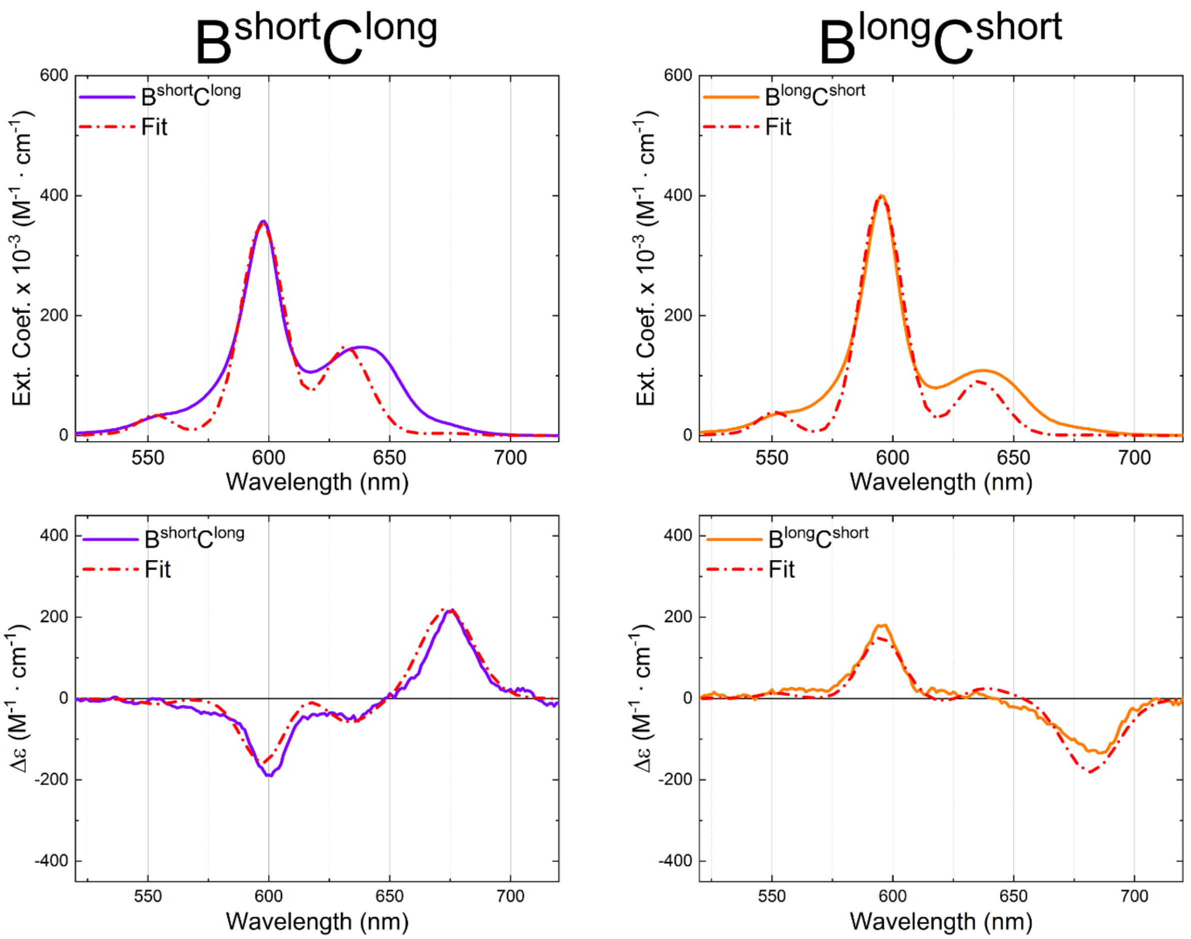

Figure S15. Acquired steady-state absorption and CD spectra (solid line) of squaraine dimers with unequal short and long linker lengths covalently templated by DNA HJ with corresponding KRM Fit (dash-dot line). The spectra were recorded at room temperature with the squaraine-DNA construct concentration 1.5 μM in 1× TBE, 15 mM $MgCl_2$. The absorption spectra were converted to the molar extinction. The CD spectra were converted to molar CD.

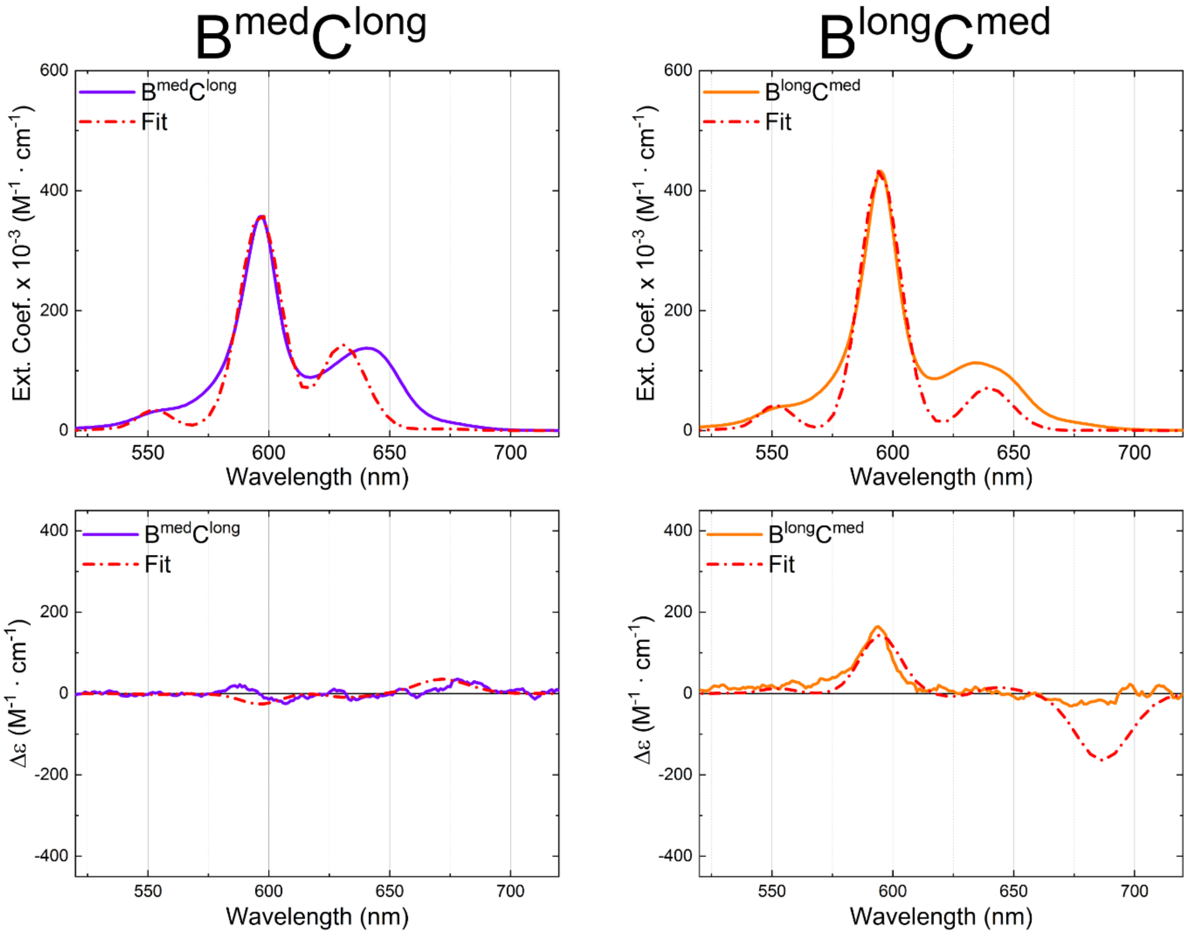

Figure S16. Acquired steady-state absorption and CD spectra (solid line) of squaraine dimers with unequal medium and long linker lengths covalently templated by DNA HJ with corresponding KRM Fit (dash-dot line). The spectra were recorded at room temperature with the squaraine-DNA construct concentration 1.5 μM in 1× TBE, 15 mM $MgCl_2$. The absorption spectra were converted to the molar extinction. The CD spectra were converted to molar CD.

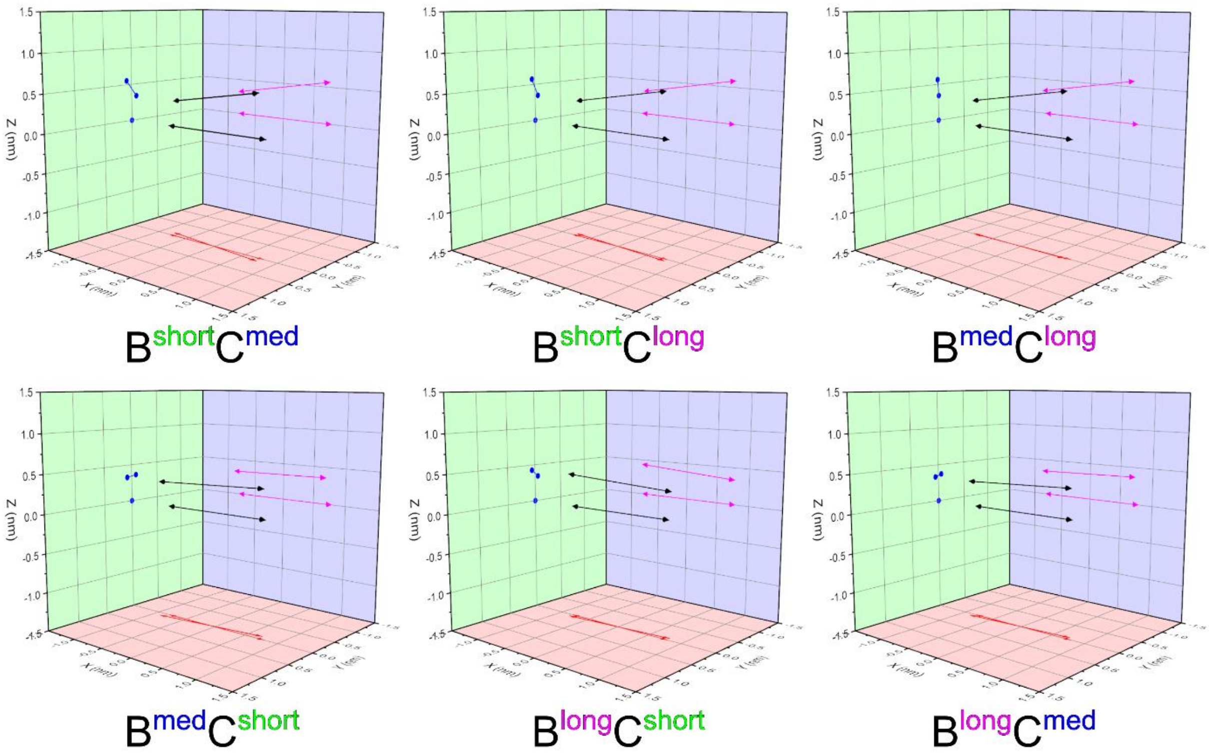

Figure S17. Three-dimensional vector plots of the KRM squaraine dimer positions with unequal linker lengths show the different plane projections XY, XZ, and YZ.

Supporting Information 7: *References*

(1) Gottlieb, H. E.; Kotlyar, V.; Nudelman, A. NMR Chemical Shifts of Common Laboratory Solvents as Trace Impurities. *J. Org. Chem.* **1997,** *62*, 7512-7515 DOI:10.1021/jo971176v.

(2) Terpetschnig, E.; Lakowicz, J. R. Synthesis and Characterization of Unsymmetrical Squaraines: A New Class of Cyanine Dyes. *Dyes Pigm.* **1993,** *21*, 227-234 DOI:10.1016/0143-7208(93)85016-S.

(3) Mass, O. A.; Wilson, C. K.; Barcenas, G.; Terpetschnig, E. A.; Obukhova, O. M.; Kolosova, O. S.; Tatarets, A. L.; Li, L.; Yurke, B.; Knowlton, W. B., et al. Influence of Hydrophobicity on Excitonic Coupling in DNA-Templated Indolenine Squaraine Dye Aggregates. *J. Phys. Chem. C* **2022**, DOI:10.1021/acs.jpcc.1c08981.

(4) Abramavicius, D.; Palmieri, B.; Mukamel, S. Extracting Single and Two-Exciton Couplings in Photosynthetic Complexes by Coherent Two-Dimensional Electronic Spectra. *Chem. Phys.* **2008,** *357*, 79-84 DOI:10.1016/j.chemphys.2008.10.010.

(5) Abramavicius, D.; Mukamel, S. Exciton Dynamics in Chromophore Aggregates with Correlated Environment Fluctuations. *J. Chem. Phys.* **2011,** *134*, 174504 DOI:10.1063/1.3579455.

(6) Abramavicius, D.; Palmieri, B.; Voronine, D. V.; Šanda, F.; Mukamel, S. Coherent Multidimensional Optical Spectroscopy of Excitons in Molecular Aggregates; Quasiparticle Versus Supermolecule Perspectives. *Chem. Rev.* **2009,** *109*, 2350-2408 DOI:10.1021/cr800268n.

(7) Czikklely, V.; Forsterling, H. D.; Kuhn, H. Extended Dipole Model for Aggregates of Dye Molecules. *Chem. Phys. Lett.* **1970,** *6*, 207-210 DOI:10.1016/0009-2614(70)80220-2.

(8) Holstein, T. Studies of Polaron Motion: Part I. The Molecular-Crystal Model. *Ann. Phys.* **1959,** *8*, 325-342 DOI:10.1016/0003-4916(59)90002-8.

(9) Chung, P.-H.; Tregidgo, C.; Suhling, K. Determining a Fluorophore's Transition Dipole Moment from Fluorescence Lifetime Measurements in Solvents of Varying Refractive Index. *Methods Appl. Fluoresc.* **2016,** *4*, 045001 DOI:10.1088/2050-6120/4/4/045001.
